# Multidrug resistance patterns and carbapenemase production among Gram-negative bacteria causing healthcare-associated infections in hospitalized patients at the University of Gondar Comprehensive Specialized Hospital, Northwest Ethiopia

**DOI:** 10.1371/journal.pone.0344280

**Published:** 2026-03-05

**Authors:** Kindu Alem, Mucheye Gizachew, Mulat Dagnew, Worku Ferede, Solomon Belay, Baye Gelaw, Feleke Moges

**Affiliations:** 1 Department of Medical Microbiology, School of Biomedical and Laboratory Sciences, College of Medicine and Health Sciences, University of Gondar, Gondar, Ethiopia; 2 Department of Biology, College of Natural and Computational Sciences, Woldia University, Woldia, Ethiopia; 3 Medical Microbiology Laboratory, University of Gondar Comprehensive Specialized Hospital, Gondar, Ethiopia; Debre Markos University, ETHIOPIA

## Abstract

**Background:**

*Klebsiella pneumoniae*, *Acinetobacter* species, and *Pseudomonas aeruginosa* are priority pathogens identified by the World Health Organization that have emerged as major causes of healthcare-associated infections. Their increasing resistance to multiple antimicrobial agents poses significant challenges to clinical management and infection control efforts.

**Objective:**

This study aimed to determine the prevalence, associated risk factors, antimicrobial resistance patterns, and carbapenemase production of *K. pneumoniae*, *Acinetobacter* spp., and *P. aeruginosa* among hospitalized patients with suspected bloodstream, urinary tract, and surgical site healthcare-associated infections at the University of Gondar Comprehensive Specialized Hospital, Northwest Ethiopia.

**Methods:**

A hospital-based cross-sectional study was conducted from August 2024 to June 2025 among 477 patients suspected of bloodstream, urinary tract, or surgical site healthcare-associated infections. Socio-demographic and clinical data were collected using a semi-structured questionnaire. Blood, urine, and wound/pus specimens were aseptically collected and inoculated on MacConkey, blood, and cysteine lactose electrolyte-deficient agar following standard microbiological techniques. Antimicrobial susceptibility testing was performed using the Kirby-Bauer disc diffusion method on Mueller-Hinton agar according to Clinical and Laboratory Standards Institute guidelines. Data were analyzed using SPSS version 27. Bivariate and multivariate logistic regression analyzes were used to identify factors associated with healthcare-associated infections. P value < 0.05 was considered statistically significant.

**Results:**

Among the 477 patients, 118 (24.7%) developed healthcare-associated infections caused by *K. pneumoniae*, *Acinetobacter* spp., and *P. aeruginosa*, with culture positivity rates of 14.9%, 4.8%, and 5%, respectively. Significant associated factors included age under five (AOR = 13.260, p < 0.001) and 5–17 (AOR = 4.081, p < 0.025), prior hospital admission (AOR = 8.302, p < 0.001), prolonged hospital stay (AOR = 3.213, p < 0.001), and admission to the orthopedic ward (AOR = 6.071, p < 0.003). Multidrug resistance was detected in 94.4% of *K. pneumonia*e, 69.6% of *Acinetobacter* spp., and 58.3% of *P. aeruginosa* isolates. Carbapenemase production occurred in 92%, 77.8%, and 57.1% of these carbapenem-resistant isolates, respectively. Amikacin, meropenem, and ciprofloxacin were the most effective antimicrobials, whereas chloramphenicol was effective only against *K. pneumonia*e.

**Conclusion:**

This study showed high prevalence of multidrug resistance and carbapenemase production among *K. pneumoniae*, *Acinetobacter* spp., and *P. aeruginosa* in the study area, highlighting the urgent need to strengthen infection prevention and control measures and to promote antimicrobial stewardship programs.

## Introduction

Antimicrobial resistance (AMR) is a worldwide crisis that jeopardizes numerous achievements of contemporary medicines. It has been called a silent pandemic, posing a risk of claiming 10 million lives annually by the year 2050 if effective actions are not implemented [1]. At present, the ESKAPE (*Enterococcus faecium*, *Staphylococcus aureus*, *Klebsiella pneumoniae*, *Acinetobacter baumannii*, *Pseudomonas aeruginosa*, and *Enterobacter* species) group of bacteria exhibit multidrug resistance, making them a significant global challenge for public health [2]. The risk is significant, leading to an estimated five million deaths globally in 2019. Carbapenem-resistant *K. pneumoniae*, *A. baumannii*, and *P. aeruginosa* are three of the six main bacterial pathogens in these deaths related to AMR [3].

The World Health Organization (WHO) classifies carbapenem-resistant *K. pneumoniae*, *A. baumannii*, and *P. aeruginosa* as pathogens of priority concern. *K. pneumoniae* and *A. baumannii* are classified as critical-priority and *P. aeruginosa* as high-priority pathogens by the WHO [4]. Infections caused by these multidrug-resistant (MDR) bacteria usually do not respond to conventional antimicrobials and result in long-term hospitalization and a higher risk of mortality, which are the main causes of healthcare-associated infections (HAIs). Healthcare-associated infections are infections that patients acquire while receiving treatment in healthcare settings, including hospitals and clinics. These infections develop 48 hours or more after admission and are not present or incubating at the time of hospital admission [5].

The most frequently reported HAIs, including bloodstream infections (BSIs), urinary tract infections (UTIs), and surgical site infections (SSIs), are commonly caused by β-lactamase-producing *K. pneumoniae* and non-fermenting Gram-negative *A. baumannii* and *P. aeruginosa*. These bacteria developed resistance to various antibiotics, posing a risk in healthcare facilities, causing sporadic outbreaks, and creating significant challenges for global healthcare management for which new antibiotic therapy is urgently required [6,7]. Healthcare-associated infections significantly endanger patient safety and overall health outcomes globally, with an even greater public health burden in low- and middle-income countries [8,9].

Hospitalized patients can acquire the MDR bacteria from both external and internal sources, mainly through direct or indirect contact with contaminated objects, invasive medical devices, other patients, healthcare workers, or visitors [10]. Several studies have identified key risk factors for HAIs, including socio-demographic characteristics such as age, sex, and place of residence, as well as clinical factors like prolonged hospitalization, previous antimicrobial-taking history, and the specific hospital ward in which the patient is admitted [11–13]. There is a critical need to explore the growing threat of AMR worldwide, particularly in Africa, where the emergence of global priority pathogens has been increasingly linked to HAIs.

The prevalence of HAIs shows considerable variation globally. In developed countries, it typically ranges from 3.5% to 12%, whereas in low- and middle-income countries, the rates can reach between 5.7% and 19.1% [14]. A systematic review and meta-analysis reported HAI prevalence rates of 12.9% in Sub-Saharan Africa and 16.96% in Ethiopia [15,16]. However, there is currently a scarcity of published data from the study area on the prevalence of specific HAIs, including BSIs, UTIs, and SSIs. Moreover, there are limited reports on the prevalence and AMR profiles in *K. pneumoniae*, *Acinetobacter* spp., and *P. aeruginosa* in Northwest Ethiopia. Therefore, the aim of this study was to determine the prevalence and AMR patterns and to identify associated risk factors for *K. pneumoniae*, *Acinetobacter* spp., and *P. aeruginosa* among hospitalized patients suspected of BSIs, UTIs, or SSIs associated with HAIs at the University of Gondar Comprehensive Specialized Hospital (UoGCSH), Northwest Ethiopia.

## Materials and methods

### Study design, area, and period

A hospital-based cross-sectional study was conducted at UoGCSH, Northwest Ethiopia, from August 2024 to June 2025, among hospitalized patients suspected of having healthcare-associated BSIs, UTIs, or SSIs. University of Gondar Comprehensive Specialized Hospital is one of the specialized teaching university hospitals. It is found in the Amhara National Regional State, located in Gondar City, which is 738 km from Addis Ababa, the capital city of Ethiopia. The hospital has been serving as the referral center for private and other government hospitals in the region. It has specialty services including internal medicine, pediatrics, surgery, gynecology, intensive care units (ICUs), obstetrics, optometry, oncology, fistula, Kaalzar, and psychiatry [17]. Now, the hospital serves more than 5 million people in the catchment area [18].

### Source and study population

All hospitalized patients of all age groups at UoGCSH were the source population. The study population included patients admitted during the study period who were clinically suspected of having healthcare-associated BSIs, UTIs, or SSIs.

### Inclusion and exclusion criteria

All age group patients hospitalized at UoGCSH with clinical evidence of healthcare-associated BSIs, UTIs, or SSIs after 48 hours of hospitalization were included, and those who had been hospitalized for less than 48 hours were excluded from the study.

### Sample size determination and sampling techniques

The sample size was determined based on the single population proportion formula: N = (Zα/2)^2^ x P x (1-P)/d^2^, where N = sample size; Z = 95% statistic for level of confidence (1.96); p = prevalence of *K. pneumoniae*, *Acinetobacter* spp., and *P. aeruginosa* (p = 23.7%) estimated from a previous study conducted in Ethiopia [19]; and d = the margin of error taken as 4%. After adding a 10% non-response rate, the estimated sample size was 477. The study employed a consecutive sampling technique, enrolling all eligible participants who met the inclusion criteria in sequence until the targeted sample size was reached.

### Data collection procedures

According to the criteria set forth by the European Centre for Disease Prevention and Control [20], all age groups of patients hospitalized in various wards of UoGCSH were monitored prospectively and evaluated by physicians for the development of bloodstream, urinary tract, or surgical site HAIs. After written informed consent and assent were obtained from the study participants, socio-demographic information (sex, age, and residence) as well as potential risk factors of HAIs was collected from each patient by face-to-face interview using a semi-structured questionnaire. Clinical data related to date of admission, admission ward type, chronic diseases, length of hospital stay, and previous antimicrobial taking history were collected by reviewing the patient’s medical record. For children, the respective guardians were interviewed.

### Blood samples collection and processing

Clinical samples, including blood, urine, and wound/pus, were collected from patients admitted to various wards of UoGCSH who were clinically suspected of having BSIs, UTIs, or SSIs. Following standard protocols, venous blood samples of 10 mL from adults, 2 mL from children, and 1 mL from neonates suspected of BSIs were collected. Patient demographic data, specimen type, ward of admission, and other relevant information were recorded on a standardized bacteriology request form by the clinicians. The collected blood samples were immediately inoculated into 5–10 mL tryptic soya broth medium (Oxoid, England) and transported without delay to the Medical Microbiology Laboratory at the University of Gondar. The inoculated bottles were incubated aerobically at 37°C and inspected daily for visible bacterial growth. Gram staining was performed on blood culture bottles that showed growth, and the positive samples were sub-cultured onto blood agar (BA) and MacConkey (MAC) agar plates (HiMedia^TM^, India). These plates were incubated aerobically at 37°C and examined after 24 hours. Blood culture bottles without visible growth were continuously monitored for up to 7 days, and those showing no growth during this period were reported as culture-negative [21].

### Urine samples collection and processing

Clean-catch midstream urine samples were collected from patients clinically suspected of UTIs. For catheterized patients, 10 mL of urine was aseptically collected into a sterile container after cleansing the catheter outlet. For non-catheterized patients, the same volume of urine was self-collected using a sterile container under proper instructions. Patient demographic data, specimen type, ward of admission, and other relevant information were recorded on a standardized bacteriology request form by the clinicians. All urine samples were immediately transported to the Medical Microbiology Laboratory of the University of Gondar. Upon arrival, 1 μL of each urine sample was inoculated onto cysteine lactose electrolyte-deficient (CLED) agar and MacConkey agar plates (HiMedia^TM^, India). Plates were incubated aerobically at 37°C for 24–48 hours and observed for bacterial growth. Colonies grown on CLED agar were counted using a colony counter, and counts of ≥10^2^ CFU/mL in catheterized patients and ≥10^5^ CFU/mL in non-catheterized patients in CLED were considered significant bacteriuria [22].

### Wound/pus samples collection and processing

Wound/pus samples were collected aseptically from each participant using sterile cotton swabs and transferred into sterile test tubes containing 0.5 mL normal saline following the Levine method [23]. Patient demographic data, specimen type, and admission ward were recorded in the standardized bacteriology request forms by clinicians. Samples were immediately transported to the Medical Microbiology Laboratory of the University of Gondar and inoculated onto BA and MAC agar plates. The plates were incubated aerobically at 35–37°C for 24–48 hours, and positive cultures were identified based on colony characteristics.

### Bacterial isolation and identification

Preliminary identification of *K. pneumoniae*, *Acinetobacter* spp., and *P. aeruginosa* was carried out based on colony morphology, Gram staining, and a panel of biochemical tests. Colonies with distinct morphology and coloration were selected and sub-cultured onto fresh MAC plates to obtain pure culture isolates.

### Biochemical tests

*K. pneumoniae* isolates were identified by using different biochemical tests, including triple-sugar iron, urease, citrate, indole, lysine decarboxylase, and motility tests. For the identification of *Acinetobacter* spp. and *P. aeruginosa,* oxidase strip tests were done in addition to the above-mentioned tests.

### Antimicrobial susceptibility testing

Antimicrobial susceptibility testing was performed using the Kirby-Bauer disc-diffusion method on the Muller Hinton agar (MHA) (HiMedia^TM^, India) to determine antimicrobial susceptibility patterns of the isolates and to interpret the results. From each isolate, 3–5 pure colonies were selected and suspended in normal saline. The turbidity of the bacterial suspension was adjusted to 0.5 McFarland standards. After 15 minutes, a sterile cotton swab was dipped into the suspension, and the inoculum was evenly spread over the entire surface of MHA. Antimicrobial impregnated paper discs of amoxicillin-clavulanate (20/10 μg), piperacillin-tazobactam (100/10 μg), piperacillin (100 μg), cefepime (30 μg), cefotaxime (30 μg), ceftriaxone (30 μg), ceftazidime (30 μg), meropenem (10 μg), imipenem (10 μg), tobramycin (10 μg), amikacin (30 μg), gentamicin (10 μg), tetracycline (30 μg), ciprofloxacin (5 μg), co-trimoxazole (1.25/23.75 μg), and chloramphenicol (30 μg) (Oxoid, England) were used for isolated pathogenic bacteria to assess their resistance patterns*.* This study followed the Clinical and Laboratory Standards Institute (CLSI, 2024) guidelines, and isolates resistant to at least one agent in three or more antimicrobial classes were considered MDR [24,25].

### Phenotypic screening of carbapenemase production

The modified carbapenem inactivation method (mCIM) was employed to screen carbapenemase production in *K. pneumoniae* and *P. aeruginosa* isolates exhibiting resistance to meropenem [24]. For this assay, 1 μL loopful of *K. pneumoniae* and 10 μL loopful of *P. aeruginosa* were each added to separate tubes containing 2 mL of tryptic soy broth (TSB) (Oxoid, England) and emulsified for 10–15 seconds. A 10 μg meropenem disc was aseptically immersed into each suspension, which was incubated for 4 hours ± 15 minutes at 37°C in ambient conditions. Meanwhile, a 0.5 McFarland suspension of the quality control strain *E. coli* ATCC 25922 was prepared in saline and uniformly inoculated onto MHA plates. After incubation, the meropenem discs were removed from the TSB and placed onto the MHA plate inoculated with the *E. coli* ATCC25922 indicator strains, followed by incubation in an inverted position for 18–24 hours at 37°C. Results were interpreted based on inhibition zones: a zone diameter of 6–15 mm, or colonies appearing within a 16–18 mm zone, indicated a positive result, whereas a zone ≥19 mm indicated a negative result [24].

For *Acinetobacter* spp. isolates, the simplified carbapenem inactivation method (sCIM) was applied. In this assay, a 0.5 McFarland standard suspension of *E. coli* ATCC 25922 was diluted 1:10 in saline and evenly spread onto MHA plates following the standard disc diffusion protocol. A 10 μg imipenem disc was then smeared with 1–3 colonies of *Acinetobacter* spp. grown overnight on BA. The smeared disc, with the inoculated side facing down, was immediately placed onto the MHA plate seeded with *E. coli* ATCC 25922. An imipenem disc without smearing served as the negative control. Plates were incubated at 37°C for 16–18 hours [26]. Carbapenemase-producing isolates hydrolyze imipenem, resulting in uninhibited growth of the indicator strain. Results were interpreted as follows: a zone of inhibition measuring 6–20 mm or satellite growth of *E. coli* colonies within ≤22 mm indicated a positive result, whereas a zone ≥26 mm was considered negative [26].

### Data quality assurance

Data collectors were trained on proper sample collection and questionnaire administration, and the questionnaire was pre-tested to ensure clarity, consistency, and reliability. All culture media were prepared according to the manufacturers’ instructions. The sterility of prepared culture media was verified by incubating 5% of the batch at 37°C overnight before using it. Sample collection, bacterial isolation, identification, and antimicrobial susceptibility testing were conducted under strict aseptic conditions. The performance of the media and the potency of antimicrobial discs were tested using American Type Culture Collection (ATCC) standard reference strains *E. coli* ATCC25922 and *P. aeruginosa* ATCC27853. For antimicrobial susceptibility testing, bacterial inoculum suspensions were standardized to a 0.5 McFarland turbidity standard.

### Data analysis and interpretation

The data were entered and coded in Microsoft Excel and then transferred to SPSS version 27.0 software for analysis. Descriptive statistics were used to present the results in frequency and percentages. Binary logistic regression was performed to determine the association between dependent and independent variables. Variables with a p-value < 0.25 in the bivariate analysis were included in the multivariate logistic regression model. Variables with a p-value of < 0.05 and an adjusted odds ratio (AOR) whose 95% confidence interval (CI) did not cross 1 in the multivariate analysis were considered statistically significant.

### Ethical approval and consent to participate

The protocol was ethically approved by the College of Medicine and Health Sciences, University of Gondar ethical review committee with protocol reference number CMHSSH-UOG IRERC/18/14/2024. Written informed consent was obtained from each study participant, and for children, assent was obtained from their legal guardians. For study participants whose samples tested positive, the results were reported to the attending clinicians.

## Results

### Socio-demographic and clinical characteristics of study participants

In this study, a total of 477 patients presenting with clinically suspected BSI, UTI, or SSI were enrolled. Among the study participants, the majorities were males, accounting for 290 (60.8%). The largest proportion, 216 (45.3%), were children under five years of age, and 273 (57.2%) were living in urban areas. Most participants, 372 (78%), had no history of prior hospital admission, while 439 (92%) had a history of antimicrobial use. In addition, 389 (81.6%) had a history of using invasive medical devices during their treatment. A considerable number of study participants, 172 (36%), were admitted to the ICU, while the majority, 387 (81.1%), had no underlying chronic diseases. Additionally, 209 (43.8%) of the admitted patients had a hospital stay of more than five days, as shown in Table 1.

**Table 1:** Socio-demographic and clinical characteristics of patients suspected of having bloodstream, urinary tract, or surgical site HAIs among hospitalized patients at UoGCSH, Gondar, Northwest Ethiopia.

Demographic and clinical variables		Frequency	Percentage (%)
Sex	Male	290	60.8
	Female	187	39.2
Age in years	< 5	216	45.3
	5-17	57	12
	18-64	169	35.4
	> 64	35	7.3
Residence	Rural	204	42.8
	Urban	273	57.2
History of previous admission	Yes	105	22.0
	No	372	78.0
Previous antibiotic taking history	Yes	439	92
	No	38	8
Invasive medical devices	Yes	389	81.6
	No	88	18.4
Underlying chronic diseases	Renal disease	23	4.8
	Cancer	26	5.5
	Hypertension	12	2.5
	Diabetes mellitus	7	1.5
	Cardiac disease	22	4.6
	None	387	81.1
Admission ward	Medical ward	80	16.8
	Surgical ward	42	8.8
	ICU	172	36
	Pediatric ward	130	27.3
	Orthopedic ward	28	5.9
	Trauma ward	11	2.3
	Other	14	2.9
Duration of hospitalization	2-5 days	268	56.2
	> 5 days	209	43.8

HAIs, healthcare-associated infections; UoGCSH, University of Gondar Comprehensive Specialized Hospital; ICU, intensive care unit

### Isolation rate of *K. pneumoniae*, *Acinetobacter* spp., and *P. aeruginosa*

Among the 477 clinical samples collected, 24.7% (118/477; 95% CI: 20.7–28.9) of *K. pneumoniae*, *Acinetobacter* spp., and *P. aeruginosa* were isolated from blood, urine, and wound/pus samples. *K. pneumoniae* was the most frequently isolated pathogen, with a prevalence of 14.9% (71/477; 95% CI: 11.7–18.1), while *Acinetobacter* spp. and *P. aeruginosa* were identified in 4.8% (23/477; 95% CI: 2.9–6.7) and 5.0% (24/477; 95% CI: 3.1–7.0) of samples, respectively. The proportions of HAIs were 63 (53.4%), 12 (10.2%), and 43 (36.4%) for BSI, UTI, and SSI, respectively. The majority of *K. pneumoniae* and *Acinetobacter* spp., 44 (62%) and 14 (60.9%), respectively, were isolated from blood samples, whereas most *P. aeruginosa* isolates, 16 (66.7%), were recovered from wound/pus samples (Table 2).

**Table 2:** Isolation rate of *K. pneumoniae*, *Acinetobacter* spp., and *P. aeruginosa* from clinical samples of hospitalized patients suspected of having bloodstream, urinary tract, or surgical site HAIs at UoGCSH, Gondar, Northwest Ethiopia.

Clinical samples	Bacteria isolated from different infection sites (n (%))			Total isolates
	*K. pneumoniae*	*Acinetobacter spp.*	*P. aeruginosa*	
Blood	44 (62)	14 (60.9)	5 (20.8)	63 (53.4)
Urine	8 (11.3)	1 (4.3)	3 (12.5)	12 (10.2)
Wound/pus	19 (26.7)	8 (34.8)	16 (66.7)	43 (36.4)
**Total**	**71 (100)**	**23 (100)**	**24 (100)**	**118 (100)**

HAIs, healthcare-associated infections; UoGCSH, University of Gondar Comprehensive Specialized Hospital; n (%), number and percentage

### Factors associated with healthcare-associated infections

The independent associated risk factors, including age, history of previous admission, duration of hospital stay, and admission ward, were identified as predictors of HAIs caused by *K. pneumoniae*, *Acinetobacter* spp., and *P. aeruginosa.* Patients under five years of age (AOR: 13.260, 95% CI: 4.320–40.698, P < 0.001) and 5–17 years of age (AOR: 4.081, 95% CI: 1.189–14.003, P < 0.025) had a statistically significant association with developing HAIs compared to older age groups. Likewise, patients with a history of prior admission (AOR: 8.302, 95% CI: 4.255–16.198, p < 0.001), patients hospitalized for more than five days (AOR: 3.213, 95% CI: 1.703–6.062, p < 0.001), and those patients admitted to the orthopedics ward (AOR: 6.071, 95% CI: 1.877–19.640, p < 0.003) were more likely to develop HAIs compared to their counterparts (Table 3).

**Table 3:** Association of independent variables with *K. pneumoniae*, *Acinetobacter* spp., and *P*. *aeruginosa* among hospitalized patients suspected of having bloodstream, urinary tract, or surgical site HAIs at UoGCSH, Gondar, Northwest Ethiopia.

Demographic and clinical variables		K. *pneumoniae*, *Acinetobacter* spp., and *P. aeruginosa*		P-value	COR with 95% CI	P-value	AOR with 95% CI
		Positive	Negative				
Sex	Male	66	224	1			
	Female	52	135	0.213	1.307 (0.858-1.993)		
Age in year	< 5	68	148	0.174	1.838 (0.765-4.416)	0.001	13.260 (4.320-40.698)
	5-17	11	46	0.934	0.957 (0.332-2.755)	0.025	4.081 (1.189-14.003)
	18-64	32	137	1		1	
	> 64	7	28	0.884	0.934 (0.375-2.329)	0.589	0.740 (0.248-2.207)
Residence	Rural	44	160	1		1	
	Urban	74	199	0.166	1.352 (0.882-2.073)	0.856	0.952 (0.561-1.616)
History of previous admission	No	65	307	1		1	
	Yes	53	52	0.001	4.814 (3.018-7.678)	0.001	8.302 (4.255-16.198)
Admission ward	Medical	10	70	1		1	
	Surgical	8	34	0.336	1.647 (0.596-4.549)	0.258	1.933 (0.617-6.059)
	ICU	50	122	0.005	2.869 (1.369-6.012)	0.448	1.555 (0.497-4. 866)
	Pediatric	32	98	0.036	2.286 (1.055-4.954)	0.262	0.481 (0.134-1.729)
	Orthopedic	13	15	0.001	5.250 (1.932-14.266)	0.003	6.071 (1.877-19.640)
	Trauma	3	8	0.202	2.625 (0.596-11.568)	0.118	4.027 (0.701-23.143)
	Other	3	11	0.378	1.909 (0.453-8.044)	0.654	1.465 (0.275-7.803)
Previous antibiotic taking history	No	7	31	1			
	Yes	111	328	0.35	1.499 (0.642-3.499)		
Duration of hospital admission	2-5 days	46	222	1		1	
	> 5 days	72	137	0.001	2.536 (1.655-3.887)	0.001	3.213 (1.703-6.062)

HAIs, healthcare-associated infections; UoGCSH, University of Gondar Comprehensive Specialized hospital; AOR, adjusted odds ratio; CI, confidence interval; COR, crude odds ratio; 1, reference

### Antimicrobial susceptibility patterns

**Fig 1:**
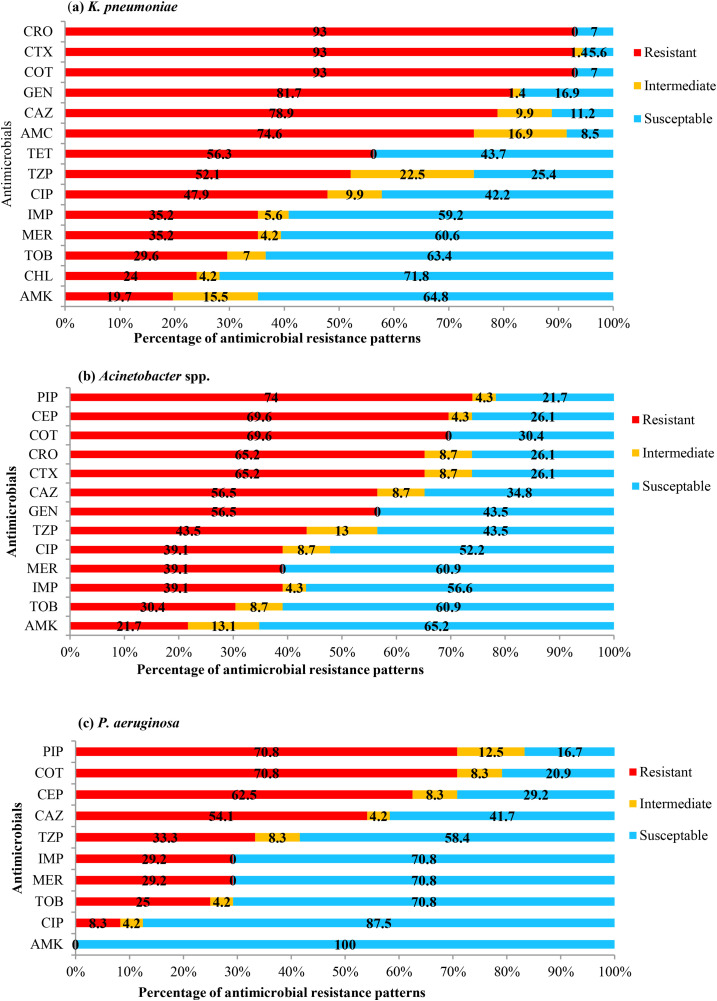
Antimicrobial resistance patterns of (a) *K. pneumoniae,* (b) *Acinetobacter* spp., and (c) *P*. *aeruginosa* among patients suspected of having HAIs at UoGCSH, Gondar, Northwest Ethiopia. HAIs, healthcare-associated infections; UoGCSH, University of Gondar Comprehensive Specialized Hospital; AMC, amoxicillin-clavulanic acid; PIP, piperacillin; TZP, piperacillin-tazobactam; CRO, ceftriaxone; CAZ, ceftazidime; CTX, cefotaxime; MER, meropenem; IMP, imipenem; AMK, amikacin; GEN, gentamicin; TOB, tobramycin; TET, tetracycline; CIP, ciprofloxacin; COT, co-trimoxazole; CHL, chloramphenicol.

Among the 71 *K. pneumoniae* isolates, 93% (66/71; 95% CI: 87.0–98.9) were resistant to ceftriaxone, cefotaxime, and co-trimoxazole, while 81.7% showed resistance to gentamicin. The lowest resistance rate, 19.7%, was observed against amikacin, followed by chloramphenicol (24%), tobramycin (29.6%), and meropenem (35.2%). Similarly, among the 23 *Acinetobacter* spp. isolates, high resistance was observed to piperacillin (74%) and cefepime (69.6%), whereas 65.2% remained susceptible to amikacin. Among the 24 *P. aeruginosa* isolates, 87.5% were susceptible to ciprofloxacin, whereas 70.8% were resistant to piperacillin and co-trimoxazole. According to 2024 CLSI guidelines, amikacin susceptibility was assessed only in three *P. aeruginosa* isolates recovered from urine specimens, and all were susceptible (100%). The overall carbapenem resistance patterns of *K. pneumoniae*, *Acinetobacter* spp., and *P. aeruginosa* were 35.2%, 39.1%, and 29.2%, respectively (Fig 1.).

### Multidrug resistance patterns and carbapenemase production

The World Health Organization-priority Gram-negative bacteria, *K. pneumoniae*, *Acinetobacter* spp., and *P. aeruginosa*, were tested against 14, 13, and 10 antimicrobial discs, respectively, to determine their multidrug-resistance patterns. Multidrug resistance was defined as non-susceptibility to at least one agent in three or more of the following nine antimicrobial classes: penicillins, β-lactam combination agents, cephems, carbapenems, aminoglycosides, tetracyclines, fluoroquinolones, folate pathway inhibitors, and phenicols, according to CLSI guidelines [24,25]. The rate of MDR was highest for *K. pneumoniae*, followed by *Acinetobacter* spp. and *P. aeruginosa*. Among the 71 *K. pneumoniae* isolates, 6 (8.5%), 21 (29.6%), 13 (18.3%), 4 (5.6%), 11 (15.5%), and 12 (16.9%) showed MDR to 3, 4, 5, 6, 7, and 8 antimicrobial classes, respectively (Table 4). Among the 23 *Acinetobacter* spp., 4 (17.4%), 2 (8.7%), 2 (8.7%), 2 (8.7%), and 6 (26.1%) showed MDR to 3, 4, 5, 6, and 7 antimicrobial classes, respectively (Table 5). While, among 24 *P. aeruginosa* isolates, 5 (20.8%), 3 (12.5%), 2 (8.3%), 3 (12.5%), and 1 (4.2%) showed MDR to 3, 4, 5, 6, and 7 antimicrobial classes, respectively (Table 6). Overall, 94.3% (67/71; 95% CI: 88.9%−99.7%) *K. pneumoniae*, 69.6% (16/23; 95% CI: 50.8–88.4) *Acinetobacter* spp., and 58.3% (14/24; 95% CI: 38.8–75.5) *P. aeruginosa* isolates were identified as MDR (Table 7).

**Table 4:** Multidrug resistance patterns of K. pneumoniae among hospitalized patients suspected of having bloodstream, urinary tract, or surgical site HAIs at UoGCSH, Gondar, Northwest Ethiopia.

No. of antimicrobial classes	No. of isolate n (%)	Antibiogram patterns	No. of distinct patterns
Resistant to none	2 (2.8)	–	2
Resistant to one	0	–	–
Resistant to two	2 (2.8)	TZP–COT	1
		CRO–CAZ–CTX–TET	1
Resistant to three	6 (8.5)	TZP–TET–COT	1
		MER–IMP–TET–COT	2
		CRO–CAZ–CTX–GEN–COT	2
		AMC–CRO–CTX–AMK–GEN	1
Resistant to four	21 (29.6)	AMC–CRO–CTX–GEN–COT	5
		CRO–CAZ–CTX–TET–CIP–COT	1
		AMC–CRO–CAZ–CTX–CIP–COT	1
		CRO–CAZ–CTX–AMK–TET–COT	1
		CRO–CAZ–CTX–GEN–TET–COT	1
		AMC–TZP–CRO–CTX–GEN–COT	1
		AMC–CRO–CAZ–CTX–GEN–COT	4
		AMC–CRO–CAZ–CTX–IMP–GEN	1
		AMC–TZP–CRO–CAZ–CTX–GEN–COT	5
		AMC–TZP–CRO–CAZ–CTX–AMK–GEN–COT	1
Resistant to five	13 (18.3)	CRO–CAZ–CTX–TET–CIP–COT–CHL	1
		TZP–CRO–CAZ–CTX–TET–COT–CHL	1
		TZP–CRO–CAZ–CTX–GEN–TET–COT	2
		AMC–CRO–CAZ–CTX–GEN–CIP–COT	4
		AMC–PTZ–CRO–CTX–GEN–CIP–COT	1
		AMC–CRO–CAZ–CTX–GEN–TET–COT	1
		AMC–CRO–CAZ–CTX–TET–CIP–COT	1
		AMC–CRO–CAZ–CTX–TOB–GEN–TET–COT	1
		AMC–PTZ–CRO–CAZ–CTX–MER–IMP–CIP–COT	1
Resistant to six	4 (5.6)	AMC–CRO–CAZ–CTX–GEN–TET–CIP–COT	1
		AMC–CRO–CAZ–CTX–IMP–GEN–TET–COT	1
		AMC–TZP–CRO–CAZ–CTX–GEN–TET–CIP–COT	1
		AMC–TZP–CRO–CAZ–CTX–IMP–TOB–AMK–GEN–CIP–COT	1
Resistant to seven	11 (15.5)	AMC–CRO–CAZ–CTX–GEN–TET–CIP–COT–CHL	1
		AMC–TZP–CRO–CTX–IMP–AMK–GEN–TET–CIP–COT	1
		AMC–TZP–CRO–CAZ–CTX–IMP–GEN–TET–COT–CHL	1
		AMC–TZP–CRO–CAZ–CTX–MER–IMP–GEN–TET–CIP–COT	1
		AMC–TZP–CRO–CAZ–CTX–MER–AMK–GEN–TET–CIP–COT	1
		AMC–TZP–CRO–CAZ–CTX–MER–IMP–TOB–GEN–TET–CIP–COT	4
		AMC–TZP–CRO–CAZ–CTX–MER–IMP–TOB–GEN–TET–COT–CHL	1
		AMC–TZP–CRO–CAZ–CTX–MER–IMP–TOB–AMK–GEN–TET–CIP–COT	1
Resistant to eight	12 (16.9)	AMC–CRO–CAZ–CTX–MER–TOB–GEN–TET–CIP–COT–CHL	1
		TZP–CRO–CAZ–CTX–MER–IMP–TOB–GEN–TET–CIP–COT–CHL	1
		TZP–CRO–CAZ–CTX–MER–IMP–TOB–AMK–GEN–TET–CIP–COT–CHL	1
		AMC–TZP–CRO–CAZ–CTX–MER–IMP–TOB–GEN–TET–CIP–COT–CHL	1
		AMC–TZP–CRO–CAZ–CTX–MER–IMP–TOB–GEN–TET–CIP–COT–CHL	2
		AMC–TZP–CRO–CTX–MER–IMP–TOB–AMK–GEN–TET–CIP–COT–CHL	1
		AMC–TZP–CRO–CAZ–CTX–MER–IMP–TOB–AMK–GEN–TET–CIP–COT–CHL	5
**Total MDR (≥3)**	**67 (94.4)**	–	–

MDR, multidrug-resistant; ≥ 3 stands for resistance to 3 or more antimicrobial classes; HAIs, healthcare-associated infections; UoGCSH, University of Gondar Comprehensive Specialized hospital; n (%), number and percentage; AMC, amoxicillin-clavulanic acid; PIP, piperacillin; TZP, piperacillin-tazobactam; CRO, ceftriaxone; CAZ, ceftazidime; CTX, cefotaxime; MER, meropenem; IMP, imipenem; AMK, amikacin; GEN, gentamicin; TOB, tobramycin; TET, tetracycline; CIP, ciprofloxacin; COT, co-trimoxazole; CHL, chloramphenicol

**Table 5:** Multidrug-resistant patterns of *Acinetobacter* spp. among hospitalized patients suspected of having bloodstream, urinary tract, or surgical site HAIs at UoGCSH, Gondar, Northwest Ethiopia.

No. of antimicrobial classes	No. of isolates n (%)	Antibiogram patterns	No. of distinct patterns
Resistant to none	1 (4.3)	–	1
Resistant to one	4 (17.4)	PIP	2
		CRO	1
		CAZ	1
Resistant to two	2 (8.7)	TZP–COT	1
		CIP–COT	1
Resistant to three	4 (17.4)	PIP–CEP–COT	1
		PIP–CEP–CTX–COT	1
		PIP–CRO–CAZ–CEP–CTX–COT	1
		CRO–CEP–CTX–AMK–GEN–COT	1
Resistant to four	2 (8.7)	PIP–CRO–CAZ–CEP–CTX–GEN–COT	2
Resistant to five	2 (8.7)	PIP–TZP–CRO–CAZ–CEP–CTX–GEN–CIP	1
		PIP–TZP–CRO–CAZ–CEP–CTX–TOB–GEN–COT	1
Resistant to six	2 (8.7)	PIP–TZP–CRO–CAZ–CEP–CTX–GEN–CIP–COT	1
		PIP–CRO–CAZ–CEP–CTX–MER–IMP–GEN–CIP–COT	1
Resistant to seven	6 (26.1)	PIP–TZP–CRO–CEP–CTX–MER–IMP–AMK–GEN–CIP–COT	1
		PIP–TZP–CRO–CAZ–CEP–CTX–MER–IMP–TOB–GEN–CIP–COT	4
		PIP–TZP–CRO–CAZ–CEP–CTX–MER–IMP–TOB–AMK–GEN–CIP–COT	1
Resistant to eight	0	–	–
**Total MDR (≥3)**	**16 (69.6)**	–	–

MDR, multidrug-resistant; ≥ 3 stands for resistance to 3 or more antimicrobial classes; HAIs, healthcare-associated infections; UoGCSH, University of Gondar Comprehensive Specialized hospital; n (%), number and percentage; PIP, piperacillin; TZP, piperacillin-tazobactam; CRO, ceftriaxone; CAZ, ceftazidime; CTX, cefotaxime; MER, meropenem; IMP, imipenem; AMK, amikacin; GEN, gentamicin; TOB, tobramycin; TET, tetracycline; CIP, ciprofloxacin; COT, co-trimoxazole

**Table 6:** Multidrug-resistant patterns of *P. aeruginosa* among hospitalized patients suspected of having bloodstream, urinary tract, or surgical site HAIs at UoGCSH, Gondar, Northwest Ethiopia.

No. of antimicrobial classes	No. of isolates n (%)	Antibiogram patterns	No. of distinct patterns
Resistant to none	3 (12.5)	–	3
Resistant to one	4 (16.7)	PIP	1
		COT	3
Resistant to two	3 (12.5)	PIP–COT	1
		PIP–CAZ–CEP	2
Resistant to three	5 (20.8)	PIP–CEP–COT	1
		PIP–TZP–MER–IMP	1
		PIP–CAZ–CEP–COT	3
Resistant to four	3 (12.5)	PIP–TZP–CAZ–CEP–COT	3
Resistant to five	2 (8.3)	PIP–CEP–MER–IMP–TOB–COT	1
		CAZ–CEP–MER–IMP–TOB–CIP–COT	1
Resistant to six	3 (12.5)	PIP–TZP–CAZ–CEP–MER–IMP–TOB–COT	3
Resistant to seven	1 (4.2)	PIP–TZP–CAZ–CEP–MER–IMP–TOB–CIP–COT	1
Resistant to eight	0	–	–
**Total MDR (≥3)**	**14 (58.3)**	–	–

MDR, multidrug-resistant; ≥ 3 stands for resistance to 3 or more antimicrobials from different classes; HAIs, healthcare-associated infections; UoGCSH, University of Gondar Comprehensive Specialized hospital; n (%), number and percentage; PIP, piperacillin; TZP, piperacillin-tazobactam; CAZ, ceftazidime; MER, meropenem; IMP, imipenem; AMK, amikacin; TOB, tobramycin; CIP, ciprofloxacin; COT, co-trimoxazole

**Table 7:** Overall MDR distribution among *K. pneumoniae*, *Acinetobacter* spp., and *P. aeruginosa* from hospitalized patients suspected of having bloodstream, urinary tract, or surgical site HAIs at UoGCSH, Gondar, Northwest Ethiopia.

Bacterial species	Total isolates (n)	MDR (≥3) n (%)
*K pneumoniae*	71	67 (94.4)
*Acinetobacter* spp.	23	16 (69.6)
*P. aeruginosa*	24	14 (58.3)

MDR, multidrug-resistant; ≥ 3 stands for resistance to 3 or more antimicrobial classes; HAIs, healthcare-associated infections; UoGCSH, University of Gondar Comprehensive Specialized hospital; n (%), number and percentage

Carbapenemase (CP) production was detected in 34 (82.9%) of carbapenem-resistant isolates. Among the causative agents of HAIs, *K. pneumoniae* was the predominant CP-producing pathogen, followed by *Acinetobacter* spp. and *P. aeruginosa*. Among the 25 carbapenem-resistant *K. pneumoniae* isolates, 23 (92%; 95% CI: 81.4–100) were CP producers, 6 (24%) from blood samples, 6 (24%) from urine samples, and 11 (44%) from wound/pus samples, while 2 (8%) were non-carbapenemase producers. Similarly, among the 9 carbapenem-resistant *Acinetobacter* spp. isolates, 7 (77.8%; 95% CI: 40.0–97.2) were CP producers, 3 (33.3%) from blood samples and 4 (44.4%) from wound/pus samples, while 2 (22.2%) were non-CP producers. In the case of *P. aeruginosa*, 4 (57.1%; 95% CI: 25.0–84.2) of the 7 carbapenem-resistant isolates were carbapenemase producers, 1 (14.3%) from blood samples and 3 (42.8%) from wound/pus samples, while 3 (42.9%) were non-CP producers. Among the total 41 carbapenem-resistant *K. pneumoniae*, *Acinetobacter* spp., and *P. aeruginosa* isolates, 34 (82.9%) were CP producers and 7 (17.1%) were not CP producers (Table 8).

**Table 8:** Rate of CP-producing *K. pneumoniae*, *Acinetobacter* spp., and *P. aeruginosa* among hospitalized patients suspected of having bloodstream, urinary tract, or surgical site HAIs at UoGCSH, Gondar, Northwest Ethiopia.

Clinical samples	Carpapenemase-producing isolates (n (%))						Total	
	*K. pneumoniae*		*Acinetobacter spp.*		*P. aeruginosa*			
	CP +ve	CP-ve	CP +ve	CP-ve	CP +ve	CP-ve	CP +ve	CP-ve
Blood	6 (24)	1 (4)	3 (33.3)	1 (11.1)	1 (14.3)	1 (14.3)	10 (76.9)	3 (23.1)
Urine	6 (24)	-	-	-	-	1 (14.3)	6 (85.7)	1 (14.3)
Wound/pus	11 (44)	1 (4)	4 (44.4)	1 (11.1)	3 (42.8)	1 (14.3)	18 (85.7)	3 (14.3)
**Total**	**23 (92)**	**2 (8)**	**7 (77.8)**	**2 (22.2)**	**4 (57.1)**	**3 (42.9)**	**34 (82.9)**	**7 (17.1)**

CP, carbapenemase; CP + ve, carbapenemase positive; CP-ve, carbapenemase negative; HAIs, healthcare-associated infections; UoGCSH, University of Gondar Comprehensive Specialized hospital; n (%), number and percentage

## Discussion

In the present study, the overall prevalence of *K. pneumoniae*, *Acinetobacter* spp., and *P. aeruginosa* isolated from blood, urine, and wound/pus samples of hospitalized patients was 24.7%, indicating a relatively high rate. This elevated prevalence may be attributed to antimicrobial selective pressure, biofilm development, horizontal gene transfer of resistance genes, and the ability of these pathogens to persist in the hospital environment, all of which promote their survival and transmission in healthcare settings. This result emphasizes the urgent need for strict infection control and robust antimicrobial stewardship programs to reduce MDR and HAIs. The observed prevalence was comparable to a previous study conducted in Northeast Ethiopia (17.7%) [27]. However, it was lower than the prevalence in North Ethiopia (39.9%) [28] but higher than that reported in Italy (11.8%) [29]. Variations in the reported prevalence across studies were likely influenced by differences in infection prevention and control practices, patient populations, diagnostic methods, antimicrobial prescribing patterns, and variation in sample size and study duration.

In the current study, the most frequently isolated bacteria were *K. pneumoniae*, followed by *P. aeruginosa* and *Acinetobacter* spp. The prevalence of *K. pneumoniae* was 14.9%. This result was consistent with the study reported in Saudi Arabia (22%) [30]. However, it was higher than a study previously reported at the Ethiopian Public Health Institute (8.2%) [31] and lower than the results reported from Jordan (51.5%) [32] and Romania (38.7%) [2]. This variation in *K. pneumoniae* prevalence reported across studies was likely attributed to differences in healthcare infrastructure and resource availability, sample size, patient populations and hospital settings, and duration of hospitalization.

In the current study, the prevalence of *Acinetobacter* spp. among HAIs was 4.8%. This finding was comparable to reports from Ethiopia (2.5%) [33], Bahir Dar in Ethiopia (3.8%) [34], Pakistan (2%) [35], the Ethiopian Public Health Institute (5.5%) [36], and Northeast Ethiopia (6.3%) [37]. However, it was higher than the prevalence reported from Ghana (0.54%) [38], but lower than that reported from another study in Romania (24.4%) [2] and Saudi Arabia (28%) [30]. In the current study, the prevalence of *P. aeruginosa* was 5%. This result was consistent with the findings reported in Jimma (3.8%) [39] and Saudi Arabia (12%) [30]. However, it was higher than the prevalence previously reported in studies conducted in Nepal (0.96%) [40] and Ethiopia (2%) [41], but lower than the rates reported from Debre Tabor in Ethiopia (21.3%) [42] and Spain (13.7%) [43]. The observed variation in the prevalence of *Acinetobacter* spp. and *P. aeruginosa* across studies may be attributed to differences in sample size, the immune status of the study participants, patients’ exposure to risk factors, and the empirical use of antibiotics without prior sensitivity testing.

Healthcare-associated infections could be acquired through direct or indirect contact between patients, healthcare workers, or contaminated objects within hospital environments [44]. The present study identified age of patients, previous hospitalization and duration of hospitalization, and admission ward as significant risk factors for HAIs. Similar findings have been reported in studies conducted in Ethiopia [11,33,45], the United Kingdom [46], Poland [47], and Thailand [48], where these factors were also associated with increased risk. The findings might be linked to the high rate of exposure of patients to healthcare-associated pathogens from clinical environments and poor infection prevention practices in the healthcare settings.

In this study, a high level of AMR was observed among *K. pneumoniae*, *Acinetobacter* spp., and *P*. *aeruginosa* isolates against commonly prescribed antibiotics. This finding demands immediate targeted interventions and strengthened antimicrobial stewardship to combat AMR and improve patient outcomes. *K. pneumoniae* showed particularly high resistance to ceftriaxone (93%), cefotaxime (93%), co-trimoxazole (93%), and gentamicin (81.7%), which might be due to frequent exposure to these antibiotics and inappropriate use. Similarly, cefotaxime resistance rates of about 96% in Portugal [49], 92.3% in Sudan [50], and 89.33% in Bangladesh [51] have been reported, which were comparable to the findings of the present study. In addition, a study from Gondar in Ethiopia reported 84% resistance rate to ceftriaxone [52], which was comparable to our result. Similarly, a 94.2% resistance rate reported in Sudan [50] was also consistent with the current study. However, the isolates in this study exhibited lower resistance to amikacin (19.7%), chloramphenicol (24%), tobramycin (29.6%), and meropenem (35.2%). A study conducted in Addis Ababa reported a tobramycin resistance rate of 31.3% [53], while a study from Kenya [54] reported a meropenem resistance rate of 33%, both of which were consistent with the present findings. However, other studies have reported lower meropenem resistance rates, including 9.4% in Addis Ababa [53], 17.4% in Nepal [40], and 17.5% in Gondar [52]. These differences could be due to several factors, including variations in hospital settings and infection control practices, variations in the age and health status of study participants, the use of invasive medical devices or surgical procedures that increase infection risk, and longer hospital stays, which provide more opportunities for exposure to resistant pathogens.

*Acinetobacter* spp. and *P. aeruginosa* also exhibited a high level of resistance to most antimicrobial agents. *Acinetobacter* spp. showed particularly notable resistance to piperacillin (74%), cefepime (69.6%), and co-trimoxazole (69.6%), likely due to frequent use of these antibiotics in hospitals, misuse, and self-prescription. In contrast, it showed lower resistance to amikacin (21.7%), tobramycin (30.4%), and meropenem (39.1%), this could be due to lower exposure of *Acinetobacter* spp. to these drugs. A study conducted in Bahir Dar, Ethiopia [34], reported a 100% resistance rate to piperacillin, which was slightly comparable to the rate observed in the present study. A study conducted in Kenya [54] reported that *A. baumannii* exhibited 18% resistance rate to amikacin, which was consistent with the findings of the present study. The observed variations among studies might be due to differences in AMR screening practices, patterns of antimicrobial prescription and misuse, infection prevention and control measures, and diagnostic methods across study areas.

In this study, *P. aeruginosa* showed high resistance to piperacillin (70.8%), co-trimoxazole (70.8%), and cefepime (62.5%), which may be associated with the excessive and inappropriate use of these antibiotics in the study area. In contrast, high susceptibility was observed to ciprofloxacin (87.5%) and amikacin (100%), possibly due to their less frequent use and the limited prevalence or mobility of resistance genes for these antibiotics among these pathogens. A study conducted in South Africa [55] reported resistance rates of 64.2% for piperacillin and 11.3% for ciprofloxacin, which were consistent with the findings of the current study, whereas a study from Iraq [56] reported a higher ciprofloxacin resistance rate of 66.7%. A similar study conducted in Ethiopia [34] reported a 100% resistance rate to piperacillin, which was consistent with the findings of the present study, and a 36.4% resistance rate to ciprofloxacin, which was higher than that observed in the present study. Previous studies in China [57,58] reported high amikacin susceptibility (>90%) in *P. aeruginosa* isolates, which is slightly comparable to the findings of the present study. These observed differences may be attributed to variations in antimicrobial prescription policies and frequencies, disparities in infection prevention and control measures, differences in resistance-screening methodologies, and genetic adaptations arising from inappropriate antibiotic use.

In the present study, an alarmingly high prevalence of MDR was observed among *K*. *pneumoniae* (94.4%), *Acinetobacter* spp. (69.6%), and *P. aeruginosa* (58.3%). The high prevalence of MDR in the study area likely reflects the presence of plasmid-mediated resistance genes, the ability of the pathogens to form biofilms, and sustained antimicrobial selection pressure. This highlights the need for strict infection control and antimicrobial stewardship to prevent the spread of resistant infections. The MDR rate among *K*. *pneumoniae* isolates in this study was consistent with a report from Tikur Anbessa Specialized Hospital (98.5%) [59] in Ethiopia. On the other hand, this finding was higher than studies conducted in Ethiopia, which reported prevalence of 83.5% [60] and 71.4% [61].

Multidrug-resistant and CP-producing *Acinetobacter* spp. and *P. aeruginosa* are significant public health threats in hospital settings. The proportion of MDR *Acinetobacter* spp. identified in this study was consistent with a previous study conducted in Ethiopia [62], which reported that 73.7% of *Acinetobacter* spp. isolates were MDR. However, similar studies conducted in Bahir Dar [34], Iran [63], Mexico [64], and Ethiopia [61] reported MDR prevalence of 100%, 98.7%, 86.8%, and 78%, respectively, for *A. baumannii*, which is higher than the present study. Similarly, the prevalence of MDR *P. aeruginosa* observed in this study aligns with previous findings reported from Ethiopia (58.9%) [62]. In contrast, a systematic review and meta-analysis conducted in Ethiopia [65] reported a pooled MDR prevalence of 80.5%, while a study from Iran [63] reported a prevalence of 68.0%, and a study from Bair Dar [34] reported 100%. All of these results were higher than the prevalence observed in this study. The variation in MDR prevalence between the present study and similar studies conducted elsewhere may be attributed to differences in the types of antimicrobials tested across the studies, as well as differences in the implementation of antimicrobial stewardship programs and infection prevention and control measures. The high MDR rates observed in these Gram-negative bacteria are associated with various antimicrobial resistance mechanisms, including β-lactamase enzymes (extended-spectrum β-lactamases and carbapenemases), aminoglycoside-modifying enzymes, efflux pumps, and alterations in permeability caused by porin or lipopolysaccharide expression defects [66].

Reports on the rate of carbapenemase production among *K. pneumoniae*, *Acinetobacter* spp., and *P. aeruginosa* isolates in Ethiopia remain limited. In the current study, out of 25 carbapenem-resistant *K*. *pneumoniae*, 23 (92%) were phenotypically confirmed as carbapenemase producers. Similarly, a study conducted in Addis Ababa, Ethiopia [67], and Sudan [50], reported carbapenemase production rates of 71.8% and 78%, respectively, which were lower than the rates observed in the present study.

Among the 9 carbapenem-resistant *Acinetobacter* spp. isolates identified in this study, 7 (77.8%) were confirmed phenotypically as carbapenemase producers. A study conducted in Northeast Ethiopia [27] reported that 5 (50%) of 10 carbapenem-resistant *Acinetobacter* spp. isolates were carbapenemase producers, while another study in Ethiopia reported a prevalence of 53.2% [68], both of which were lower than the findings of the present study. A similar study conducted in Sudan [50] reported a higher carbapenemase production rate of 89%, exceeding the prevalence observed in the present study. In the present study, out of 7 carbapenem-resistant *P. aeruginosa* isolates, 4 (57.1%) were phenotypically confirmed as carbapenemase producers. In comparison, a study conducted in Northeast Ethiopia [27] reported that 14 (73.7%) of 19 carbapenem-resistant *P. aeruginosa* isolates were carbapenemase producers, a rate higher than the results observed in the current study. Different studies reported that the differences in carbapenemase production rates may be influenced by variations in frequency and spectrum of carbapenem use across hospitals and regions, the implementation of infection prevention and control practices, the diagnostic and detection methods employed, and variation in sample size and study populations.

### Limitations of the study

Although this study holds significant public health importance, it was not without limitations. Due to its cross-sectional design, HAIs that developed after patient discharge could not be detected due to the absence of follow-up, potentially leading to an underestimation of pathogen isolation rates and HAI prevalence. In addition, pathogen identification relied solely on phenotypic characterization methods due to limited resources. Moreover, molecular testing to detect carbapenemase-encoding genes in carbapenemase-positive isolates could not be performed.

### Conclusion and recommendations

This study revealed a substantial burden of HAIs caused by WHO-priority antimicrobial-resistant pathogens, with a particularly high prevalence of MDR among *K. pneumoniae*, *Acinetobacter* spp., and *P. aeruginosa*. Age of study participants, previous hospital admission, prolonged hospital stays, and ward of admission were significant risk factors. Isolates showed high resistance to commonly used antimicrobials, with *K. pneumoniae* being the most frequently isolated. Alarmingly, carbapenem resistance was observed across all isolates. The high MDR prevalence poses a critical clinical and public health threat, emphasizing the urgent need to strengthen antimicrobial stewardship, strict infection prevention and control measures, and enhance resistance surveillance in healthcare settings.
