# Supplementary material for: Multidrug resistance patterns and carbapenemase production among Gram-negative bacteria causing healthcare-associated infections in hospitalized patients at the University of Gondar Comprehensive Specialized Hospital, Northwest Ethiopia
Source: PLoS One. 2026 Mar 5;21(3):e0344280. doi: 10.1371/journal.pone.0344280 (PMC13275099; doi:10.1371/journal.pone.0344280)
Supplement: Attachment — Submitted filename: Manuscript review for PONE-D-25-63747 Dec 31 2025.docx [file pone.0344280.s001.docx]

Manuscript title: **Multidrug-resistance patterns and carbapenemase production in major healthcare-associated infection pathogens among hospitalized patients at the University of Gondar Comprehensive Specialized Hospital, Northwest Ethiopia**

Dear editor,

In this manuscript, and based on its contest, the author raised an important issue and tried to address the most global, regional and national sensitive issues, drug resistance. The document (including the introduction, the methods, the discussion…) is well written, well organized, or structured. How ever, the manuscript has a number of issues (comments, and questions) that need to be addressed before published in the journal. Some of the most important comments and questions have been addressed in the reviewer session for the author.

Decision of the reviewer: **Minor revision**

**Comments**

**Minor**

I. As it is an agreed standard, it is better to use scientific names of short forms in the abstracts too, to reduce the number of words and space. E.g. *K. pneumonia* instead of *Klebsiella pneumonia*
II. There are a number of textual and grammatical errors, fonts. Better to check thoroughly and consider in your final submission
III. Line 34: structured questionnaire- better to say semi-structured questionnaire
IV. Line 337: Carbapenemase (CP) production was detected in 34 (82.9%) of carbapenem-resistant isolates. Please re-state, as it is not clear.
V. Line 390-391: On your discussion sensitivity testing, and inadequate adherence to antimicrobial stewardship practices [40]. Healthcare-associated infections could be acquired through direct or indirect contact between patients, healthcare workers, visitors, or contaminated objects within hospital environments [54]. As it is not your part better to remove.

**Major comments**

I. The title seems too specific or too narrow (single study site, specific bacteria…)
II. The result parts of the abstract, lacks clarity as some stated under the question session
III. Your results are good and explanatory, but it seems that you tried to include only tables. Better to consider some figures
IV. Your discussion is fine. However, it is better if you put the implications of the findings.
V. Your conclusion part: “This study revealed a 24.7% prevalence of HAIs and 82.2% rate of MDR among World Health Organization-priority antimicrobial-resistant pathogens, including K. pneumoniae, Acinetobacter spp., and P. aeruginosae.” Is not a good conclusion remark. Better to re-write.

**Questions**

1. Why did you make a multicenter study? As it was better explanatory and used for generalization.
2. What is especial for your study as there are a number of related previously conducted and published articles in the North eastern and North western Ethiopia including but not limited for:

1. Kemal M, Demeke G, Adugna A, Dilnessa T, Abebaw A, Esmael A. Prevalence, antimicrobial resistance profiles, and determinants of Acinetobacter baumannii and Pseudomonas aeruginosa isolates among nosocomial infection--suspected patients in the northwestern region of Ethiopia. American Journal of Infection Control. 2025 Mar 7.1.

2. Asmare Z, Reta MA, Gashaw Y, Getachew E, Sisay A, Gashaw M, Tamrat E, Kidie AA, Abebe W, Misganaw T, Ashagre A. Antimicrobial resistance profile of Pseudomonas aeruginosa clinical isolates from healthcare-associated infections in Ethiopia: A systematic review and meta-analysis. Plos one. 2024 Aug 13;19(8):e0308946.

3. Mekonnen H, Seid A, Molla Fenta G, Gebrecherkos T. Antimicrobial resistance profiles and associated factors of Acinetobacter and Pseudomonas aeruginosa nosocomial infection among patients admitted at Dessie comprehensive specialized Hospital, North-East Ethiopia. A cross-sectional study. Plos one. 2021 Nov 15;16(11):e0257272.

3. Why you are interested to assess only the three bacterial pathogens?
4. On your abstract, Line 44: what are the number or percentage of Carbapenem resistance pattern of the isolates?
5. For which species of bacteria that Amikacin, chloramphenicol, meropenem, and ciprofloxacin were the most effective antimicrobials?
6. In your sample size calculation, you consider the p = 23.7% from the previous study. However, as I checked, this is only for K. pneumonia. Could you generalize by taking the P from a single etiologic agent, and considering for others?
7. How could you manage for those bacteria that are intrinsically resistance in including and assessing the resistance patterns. E.g. A. baumannii?
8. In your result analysis, did you consider the carbapenem production in the assessment of MDR or exclude them?
