# Supplementary material for: Multidrug resistance patterns and carbapenemase production among Gram-negative bacteria causing healthcare-associated infections in hospitalized patients at the University of Gondar Comprehensive Specialized Hospital, Northwest Ethiopia
Source: PLoS One. 2026 Mar 5;21(3):e0344280. doi: 10.1371/journal.pone.0344280 (PMC13275099; doi:10.1371/journal.pone.0344280)
Supplement: Attachment — Submitted filename: Response to Reviewers.docx [file pone.0344280.s002.docx]

**Rebuttal Letter**

We sincerely thank and appreciate the Editor and the Reviewers for their constructive and valuable comments and for the time they devoted to reviewing our manuscript. The constructive suggestions have substantially helped us to improve the quality of the manuscript. We have carefully revised the manuscript to address all concerns given by the Editor and Reviewers on manuscript in title of “**Multidrug resistance patterns and carbapenemase production among Gram-negative bacteria causing healthcare-associated infections in hospitalized patients at the University of Gondar Comprehensive Specialized Hospital, Northwest Ethiopia”** with manuscript ID**:** PONE-D-25-63747. The authors’ responses to the comments and questions raised by the Editor and Reviewers are highlighted in yellow. Similarly, all important corrections in the manuscript are highlighted in yellow in the revised version ('Revised Manuscript with Track Changes').

**Editor comment**

1. Please ensure that your manuscript meets PLOS ONE's style requirements, including those for file naming.
2. Please amend the manuscript submission data
3. Please amend your authorship list in your manuscript file to include author Kindu Alem Molla
4. If the reviewer comments include a recommendation to cite specific previously published works, please review and evaluate these publications to determine whether they are relevant and should be cited.
5. The paper requires intensive revision in terms of grammar typographical errors.
6. Essential components such as Conflict of interest, consent for publication, availability of data and material, funding, etc. should be included in the declaration sections of the paper.
7. What has been done for positive cases for the bacteria? It should be mentioned in the ethical consideration section.
8. Properly follow the authors guide for manuscript writing of PloS One.

**Authors’ response to Editor’s comment**

1. We prepared our manuscript based on author guideline of PLOS ONE. Thus, it meets PLOS ONE's style requirements.
2. We have reviewed and amended the manuscript submission data by checking the title, author names, corresponding author details, abstracts, key words, funding information, ethics approval statement, conflict of interest declaration, and cover letter details.
3. The author Kindu Alem is included in the authorship list of the manuscript
4. The reference suggested by the reviewer has been included and appropriately cited in the manuscript.
5. The manuscript has been carefully reviewed and revised to address the grammatical and topographical errors throughout the text.
6. The manuscript has been revised with the comments given by the Editor to include conflict of interest, consent for publication, availability of data, and funding.
7. For study participants whose samples tested positive for pathogenic bacteria, the results were reported to the attending clinicians, and it is incorporated in the ethical section.
8. The manuscript has been written in strict accordance with the PLOS ONE author guidelines.

**Reviewer’s comments**

**Reviewer #1:**

1. Replace the outdated references with more recent studies. In particular, the authors may consider using the following two recent references, which are closely and directly related to the scope and findings of the present study.

**Authors’ response to reviewer #1 comments**

1. We have included the recent reference commented by the reviewer.

**Reviewer #2:**

1. Comments on Title:

Hyphen between Healthcare and associated, Healthcare-associated
• Omit hyphen from multidrug-resistance
• Please rewrite as “major pathogens causing healthcare-associated infections”
• The title seems too long.

2. What is five to seventeen? You mean years?
3. You’ve used a margin of error of 4%, but your confidence level is set at 95%, which seems contradictory. You may want to either adjust the margin of error to 5% or increase the confidence level to 96%. (Line 121)
4. *P. aeruginosa* are presented in Fig 1. Where is the figure? I didn’t see it (Line283)

**Authors’ response to reviewer #2 comments and questions**

There is already hyphen between healthcare and associated and we have removed hyphen from multidrug-resistance. We have rewritten the title as “**Multidrug resistance patterns and carbapenemase production among Gram-negative bacteria causing healthcare-associated infections in hospitalized patients at the University of Gondar Comprehensive Specialized Hospital, Northwest Ethiopia”.**

1. The age category was revised from “five to seventeen” to 5-17 years with all related analysis updatee accordingly.
2. 95% confidence level with 4% margin of error is statistically is valid and commonly used in biomedical researches.
3. Although Fig 1. is sited in the manuscript, however, it was submitted as a separate file in accordance with PLOS ONE author guidelines.

**Reviewer #3:**

I. As it is an agreed standard, it is better to use scientific names of short forms in the abstracts too, to reduce the number of words and space. E.g. *K. pneumonia* instead of *Klebsiella pneumonia*
II. There are a number of textual and grammatical errors, fonts. Better to check thoroughly and consider in your final submission.
III. Line 34: structured questionnaire- better to say semi-structured questionnaire
IV. Line 337: Carbapenemase (CP) production was detected in 34 (82.9%) of carbapenem-resistant isolates. Please re-state, as it is not clear.
V. Line 390-391: On your discussion sensitivity testing, and inadequate adherence to antimicrobial stewardship practices [40]. Healthcare-associated infections could be acquired through direct or indirect contact between patients, healthcare workers, visitors, or contaminated objects within hospital environments [54]. As it is not your part better to remove.
VI. The title seems too specific or too narrow (single study site, specific bacteria
VII. The result parts of the abstract, lacks clarity as some stated under the question session
VIII. Your results are good and explanatory, but it seems that you tried to include only tables. Better to consider some figures
IX. Your discussion is fine. However, it is better if you put the implications of the findings.
X. Your conclusion part: “This study revealed a 24.7% prevalence of HAIs and 82.2% rate of MDR among World Health Organization-priority antimicrobial-resistant pathogens, including K. pneumoniae, Acinetobacter spp., and *P. aeruginosa*.” Is not a good conclusion remark. Better to re-write.
1. Why did you make a multicenter study? As it was better explanatory and used for generalization.
2. What is especial for your study as there are a number of related previously conducted and published articles in the North eastern and North western Ethiopia
3. Why you are interested to assess only the three bacterial pathogens?
4. On your abstract, Line 44: what are the number or percentage of Carbapenem resistance pattern of the isolates?
5. For which species of bacteria that Amikacin, chloramphenicol, meropenem, and ciprofloxacin were the most effective antimicrobials?
6. In your sample size calculation, you consider the p = 23.7% from the previous study. However, as I checked, this is only for K. pneumonia. Could you generalize by taking the P from a single etiologic agent, and considering for others?
7. How could you manage for those bacteria that are intrinsically resistance in including and assessing the resistance patterns. E.g. A. baumannii?
8. In your result analysis, did you consider the carbapenem production in the assessment of MDR or exclude them?

**Authors’ response to reviewer #3 comments and questions**

I. In the abstract, the first mention of each organism uses the full scientific name (*Klebsiella pneumoniae and Pseudomonas aeruginosa*) which is recommended by most journal including PLOS ONE for clarity of readers, followed by the appropriate abbreviations (*K. pneumoniae and* *P. aeruginosa*) in subsequent mentions.
II. Textual and grammatical error, as well as font size inconsistency, have been corrected in the final manuscript. However, a small font size was used for large tables and figures, which is permitted by the PLOS ONE journal guidelines.
III. Line 34: structured questionnaire is rewritten in semi-structured questionnaire.
IV. We have included a sentence to improve clarity by specifying the total number of carbapenem-resistant isolates and clearly distinguishing carbapenemase producing from non-producing isolates.
V. In our discussion part we have not removed healthcare workers, or contaminated objects within hospital environments since the contact between health workers, patients and hospital environment plays a significant role in the transmission of healthcare associated infections in hospitalized patients.
VI. The title of the manuscript is specific which helps to clearly reflect the study setting, patient population, and pathogens investigated.
VII. We have addressed the reviewer’s comments regarding the lack of clarity on the results section of the abstract.
VIII. We have added three figures presenting antimicrobial resistance patterns of (a) *K. pneumoniae*, (b) *Acinetobacter* spp., and (c) *P. aeruginosa*. Although they are combined under a single figure title, each panel clearly represents the respective pathogen.
IX. We have addressed the implication of our findings in the discussion, emphasizing their relevance for clinical practice and infection control.
X. We have revised the conclusion part to avoid reporting specific figures and to provide a more interpretive and meaningful summary.
1. Our study was not designed as a multicenter study, rather, it was a hospital based cross-sectional study at one specialized hospital. It was clearly indicated in the methods part of the manuscript
2. There are very few studies conducted in this study are concerning *K. pneumoniae*, *Acinetobacter* spp., and *P. aeruginosa* in relation to healthcare-associated infections caused by these three bacteria, especially in the study area, Gondar. Even, the very few studies reported does not include *K. pneumoniae,* which is one of the major pathogens that cause healthcare-associated infections. This gap initiates us to focus on these three bacteria which are the major healthcare-associated infections especially in intensive care units.
3. The reason why we focused on the three bacteria, Klebsiella pneumoniae, Acinetobacter baumannii, and Pseudomonas aeruginosa, is because they are the most prevalent causes of healthcare-associated infections, and they are recognized as WHO priority pathogens due to their high level of MDR and carbapenem resistance. These pathogens also highly associated with prolonged hospitalization and limited treatment options.
4. On line 44 on abstract part, we have reported the percentage of carbapenemase production in *K. pneumoniae*, *Acinetobacter* spp., and *P. aeruginosa*. However, the percentage of carbapenem resistance was reported on the result part Figure 1 which was submitted separately during submission with carbapenem resistance pattern of *K. pneumoniae*, *Acinetobacter* spp., and *P. aeruginosa* were 35.2%, 39.1%, and 29.2%, respectively. However, we have reported on result section page 13 line 295 and 296 of the revised manuscript.
5. Amikacin, chloramphenicol, and meropenem were effective for *K.* pneumoniae. For *Acinetobacter* spp., amikacin and meropenem were effective antibiotics. In *P. aeruginosa*, amikacin, ciprofloxacin, and meropenem were effective.  Chloramphenicol was not tested against *Acinetobacter* spp. and *P. aeruginosa* because it is not recommended by CLSI guidelines for these pathogens.
6. The prevalence value of 23.7% was selected from a previous study on Klebsiella pneumoniae because it represents the **highest reported prevalence among the target pathogens** (K. pneumoniae, Acinetobacter spp., and Pseudomonas aeruginosa) in comparable settings. Applying the maximum prevalence provides or it yields a large sample size and ensures adequate power to detect resistance pattern across all included pathogens.
7. For organisms in which intrinsically resistance (e.g., *A. baumannii*), antibiotics to which the species is intrinsically resistant were excluded from resistance rate determination and MDR classification, in accordance CLSI guideline.
8. Carbapenemase production was analyzed separately as a resistance mechanism, not as MDR. MDR classification was based on antimicrobial susceptibility testing results across different antibiotic classes.
