# Supplementary material for: Multidrug resistance patterns and carbapenemase production among Gram-negative bacteria causing healthcare-associated infections in hospitalized patients at the University of Gondar Comprehensive Specialized Hospital, Northwest Ethiopia
Source: PLoS One. 2026 Mar 5;21(3):e0344280. doi: 10.1371/journal.pone.0344280 (PMC13275099; doi:10.1371/journal.pone.0344280)
Supplement: Attachment — Submitted filename: Response_to_Reviewers_auresp_2.docx [file pone.0344280.s003.docx]

**Rebuttal Letter**

We sincerely thank and appreciate the Editor and the Reviewers for their constructive and valuable comments and for the time they devoted to reviewing our manuscript. The constructive suggestions have substantially helped us to improve the quality of the manuscript. We have carefully revised the manuscript to address all concerns given by the Editor and Reviewers on manuscript in title of “**Multidrug resistance patterns and carbapenemase production among Gram-negative bacteria causing healthcare-associated infections in hospitalized patients at the University of Gondar Comprehensive Specialized Hospital, Northwest Ethiopia”** with manuscript ID**:** PONE-D-25-63747. The authors’ responses to the comments and questions raised by the Editor and Reviewers are responded accordingly. Similarly, all important corrections in the manuscript are highlighted in yellow in the revised version ('Revised Manuscript with Track Changes').

**Journal Requirements**

1. If the reviewer comments include a recommendation to cite specific previously published works, please review and evaluate these publications to determine whether they are relevant and should be cited. There is no requirement to cite these works unless the editor has indicated otherwise.

**Response:** Thank you for the comment. Reviewer 1 recommended that we consider two references, including (1) Hasan SA, Raoof WM, Ahmed KK. Antibacterial activity of deer musk and Ziziphus spina-christi against carbapebem resis-tant gram negative bacteria isolated from patients with burns and wounds. Regulatory Mechanisms in Biosystems. 2024 Apr 17;15(2):267-78. [DOI: <https://doi.org/10.15421/022439>] and (2) Hasan SA, Raoof WM, Ahmed KK. FIRST REPORT OF CO-HARBORING BLEOMYCIN RESISTANCE GENE (bleMBL) AND CARBAPENEMASE RESISTANCE GENE (blaNDM-1) KLEBSIELLA PNEUMONIAE IN IRAQ WITH COMPARISON STUDY AMONG THE SENSITIVITY TEST, THE BD PHOENIX CPO DETECT TEST, AND THE RAPIDEC® CARBA NP TEST. Siberian Journal of Life Sciences and Agriculture. 2024 Aug 31;16(4):208-37. [<https://doi.org/10.12731/2658-6649-2024-16-4-1249>]. After careful evaluation, we found that the first reference is relevant to our study and has been incorporated into the revised manuscript. However, the second reference is not directly aligned with the scope and objectives of our work.

2. Please review your reference list to ensure that it is complete and correct.

**Response:** Thank you for the comment. We have carefully reviewed and updated the reference lists to insure its completeness and correctness. References that are published before 2018 have been removed where appropriate, however, seminal or foundational studies essential to the context of our have been retained. works are included. Additionally, we confirm that no retracted articles are cited in the manuscript.

**Additional Editor Comments**

**Abstract**

- Line 24: **Background**, not introduction
- Line 28-31: The objective part lacks the main objective, ‘carbapenemase production.’ Even the general objective should be parallel with the title of the manuscript. Therefore, it requires revision.
- To which media were the specimens inoculated?
- The analysis part also should be incorporated with Bivariate/multivariate logistic regression
- Line 41: Significant associated factors included………….
- Line 48: This study showed……
- Line 52: carbapenemase production
- Lines 94 and 95: Therefore, the aim of this study was to determine the prevalence and AMR patterns and associated factors…………..

**Materials and methods**

- Line 108 and 109: Now, the hospital serves more than 5 million people in the catchment area [18].
- Please make UoGCSH rather than UGCSH throughout the document
- Line 119: **Sample size determination and sampling technique**
- Line 120: The sample size was………………..
- Line 219: **Data quality assurance**
- **Results**
- Line 256: **Isolation rate of *K. pneumoniae, Acinetobacter* spp., and *P. aeruginosa***
- Line 289 and 290: Remove the following sentence ‘The antimicrobial susceptibility patterns of the isolated *K. pneumoniae, Acinetobacter* spp., and *P. aeruginosa* are presented in Fig 1.’ and add (Fig.1) at the end of this paragraph in bracket. Again, the caption for this figure should be placed below this citation, i.e. Fig 1. Antimicrobial resistance patterns of (a*) K. pneumoniae,* (b) *Acinetobacter spp.,* and (c) *P****.*** *aeruginosa* among patients suspected of having HAIs at UGCSH, Gondar, Northwest Ethiopia.
- Line 313**:** Remove ‘The overall MDR prevalence among all isolates was 82.2% (97/118; 95% CI: 75.3-89.1)’ and also from Figure 7 of the last row. It is impossible to calculate/determine overall MDR for more than one type of bacteria.
- **References**
- The name of the journal and bacteria name should be italized.
- Line 608: CLSI. Performance Standards for Antimicrobial…………………

**Responses to Editor Comments for abstract part**

- Thank you for the comment. In the abstract part, the heading “Introduction” is replaced with “Background” as suggested.
- Thank you for the constructive comment. The objective is corrected by incorporating carbapenemase production as follows “The aim of this study was to determine the prevalence, associated risk factors, antimicrobial resistance patterns, and carbapenemase production of *K. pneumoniae*, *Acinetobacter* spp., and *P. aeruginosa* among hospitalized patients suspected of healthcare-associated infections at the University of Gondar Comprehensive Specialized Hospital, Northwest Ethiopia.”
- Thank you for the valuable comment. The specimens were inoculated onto MacConkey, blood, and cysteine lactose electrolyte-deficient agar, as clearly stated in the revised manuscript.
- Thank you for the helpful comment. Bivariate and multivariate logistic regression analyzes have been incorporated in the method section of the abstract.
- Thank you for the valuable comment. The phrase “Significant risk factors” has been revised to “Significant associated factors” as recommended.
- Thank you for the constructive comment. The phrase “our study shows” has been revised into “This study showed” as suggested.
- Thank you for the comment. The phrase “carbapenemase production” is incorporated into the conclusion section of the abstract in the revised manuscript.
- Thank you for the comment. In lines 94 and 95 of the introduction, the word “Thus” is replaced with “Therefore” as suggested.

**Responses to Editor Comments for Materials and Methods**

- Thank you for the valuable comment. In the revised manuscript, line 104 has been corrected to read: “Now, the hospital serves more than 5 million people in the catchment area”, with a comma added after “Now” as recommended.
- Thank you for the comment. All occurrence of “UGCSH” have been corrected to “UoGCSH” throughout the revised manuscript.
- Thank you for the valuable comment. On line 114 of the revised manuscript “Sample size and sampling procedures**”** has been corrected to **“Sample size determination and sampling techniques” as suggested.**
- Thank you for the comment. On line 115 of the revised manuscript, “Sample size was” has been corrected to “The sample size was” as recommended.
- Thank you for the comment. On line 210 of the revised manuscript, “Quality assurance” has been corrected to “**Data** quality assurance**” as suggested.**

**Responses to Editor Comments for Result part**

- Thank you for the comment. On line 253 of the revised manuscript, the heading “Isolation rate of *K. pneumoniae*, *Acinetobacter* spp., and *P. aeruginosa* in healthcare-associated infections” has been corrected to “Isolation rate of *K. pneumoniae, Acinetobacter* spp., and *P. aeruginosa*” as recommended.
- Thank you for the constructive comment. On lines 289 and 290 of previous unrevised manuscript, the sentence “The antimicrobial susceptibility patterns of the isolated *K. pneumoniae*, *Acinetobacter* spp., and *P. aeruginosa* are presented in Fig 1.” Has been removed and “(Fig 1) has been added at the end of the paragraph. Additionally, the figure caption has been placed below the citation as recommended. “

**Fig 1. Antimicrobial resistance patterns of (a) K. pneumoniae, (b) Acinetobacter spp., and (c) P*.* aeruginosa among patients suspected of having HAIs at UGCSH, Gondar, Northwest Ethiopia.** HAIs, healthcare-associated infections; UoGCSH, University of Gondar Comprehensive Specialized Hospital; AMC**,** amoxicillin-clavulanic acid; PIP, piperacillin; TZP, piperacillin-tazobactam; CRO, ceftriaxone; CAZ, ceftazidime; CTX, cefotaxime; MER, meropenem; IMP, imipenem; AMK, amikacin; GEN, gentamicin; TOB, tobramycin; TET, tetracycline; CIP, ciprofloxacin; COT, co-trimoxazole; CHL, chloramphenicol

- Thank you for the constructive comment. On line 313 of the unrevised manuscript, the sentence “The overall MDR prevalence among all isolates was 82.2% (97/118; 95% CI: 75.3-89.1) has been removed, and the corresponding value has also been removed from the last row of Figure 7, since calculating an overall MDR across multiple bacterial species is not appropriate.

**Responses to Editor Comments to References Part**

- Thank you for the comment. In all references, the journal names and bacterial scientific names have been italicized as required.
- Thank you for the comment. On line 608 of the previous unrevised manuscript, the reference has been corrected from “CLSI, Performance Standards for Antimicrobial Susceptibility Testing, 34th Edition. 2024. 1-382” to “CLSI. Performance Standards for Antimicrobial Susceptibility Testing, 34th Edition. 2024. 1-382” by removing the comma and adding a full stop after “CLSI” as suggested.
