# Supplementary material for: Multidrug resistance patterns and carbapenemase production among Gram-negative bacteria causing healthcare-associated infections in hospitalized patients at the University of Gondar Comprehensive Specialized Hospital, Northwest Ethiopia
Source: PLoS One. 2026 Mar 5;21(3):e0344280. doi: 10.1371/journal.pone.0344280 (PMC13275099; doi:10.1371/journal.pone.0344280)
Supplement: Peer review history

# Peer review: Decision Letter 0

**DOI:** 10.1371/journal.pone.0344280.r001

**Authors:** Tebelay Dilnessa

10 Jan 2026

PONE-D-25-63747

Multidrug-resistance patterns and carbapenemase production in major healthcare-associated infection pathogens among hospitalized patients at the University of Gondar Comprehensive Specialized Hospital, Northwest Ethiopia

PLOS One

Dear Dr. Molla,

Thank you for submitting your manuscript to PLOS ONE. After careful consideration, we feel that it has merit but does not fully meet PLOS ONE’s publication criteria as it currently stands. Therefore, we invite you to submit a revised version of the manuscript that addresses the points raised during the review process.

Please submit your revised manuscript by Feb 24 2026 11:59PM. If you will need more time than this to complete your revisions, please reply to this message or contact the journal office at plosone@plos.org. When you're ready to submit your revision, log on to https://www.editorialmanager.com/pone/ and select the 'Submissions Needing Revision' folder to locate your manuscript file.

Please include the following items when submitting your revised manuscript:

- A letter that responds to each point raised by the academic editor and reviewer(s). You should upload this letter as a separate file labeled 'Response to Reviewers'.
- A marked-up copy of your manuscript that highlights changes made to the original version. You should upload this as a separate file labeled 'Revised Manuscript with Track Changes'.
- An unmarked version of your revised paper without tracked changes. You should upload this as a separate file labeled 'Manuscript'.

If you would like to make changes to your financial disclosure, please include your updated statement in your cover letter. Guidelines for resubmitting your figure files are available below the reviewer comments at the end of this letter.

If applicable, we recommend that you deposit your laboratory protocols in protocols.io to enhance the reproducibility of your results. Protocols.io assigns your protocol its own identifier (DOI) so that it can be cited independently in the future. For instructions see: https://journals.plos.org/plosone/s/submission-guidelines#loc-laboratory-protocols. Additionally, PLOS ONE offers an option for publishing peer-reviewed Lab Protocol articles, which describe protocols hosted on protocols.io. Read more information on sharing protocols at https://plos.org/protocols?utm_medium=editorial-email&utm_source=authorletters&utm_campaign=protocols.

We look forward to receiving your revised manuscript.

Kind regards,

Tebelay Dilnessa, MSc

Academic Editor

PLOS One

Journal Requirements:

When submitting your revision, we need you to address these additional requirements.

1. Please ensure that your manuscript meets PLOS ONE's style requirements, including those for file naming. The PLOS ONE style templates can be found at

https://journals.plos.org/plosone/s/file?id=wjVg/PLOSOne_formatting_sample_main_body.pdf and

https://journals.plos.org/plosone/s/file?id=ba62/PLOSOne_formatting_sample_title_authors_affiliations.pdf

2. Please amend the manuscript submission data (via Edit Submission) to include author Kindu Alem

3. Please amend your authorship list in your manuscript file to include author Kindu Alem Molla

4. If the reviewer comments include a recommendation to cite specific previously published works, please review and evaluate these publications to determine whether they are relevant and should be cited. There is no requirement to cite these works unless the editor has indicated otherwise.

Additional Editor Comments:

- The paper requires intensive revision in terms of grammer typographical errors.

- No paragraphing was needed in the abstract.

- Essential components such as Conflict of interest, consent for publication, availability of data and material, funding, etc. should be included in the declaration sections of the paper.

- What has been done for positive cases for the bacteria? It should be mentioned in the ethical consideration section.

- Properly follow the authors guide for manuscript writing of PloS One.

[Note: HTML markup is below. Please do not edit.]

Reviewers' comments:

Reviewer's Responses to Questions

**Comments to the Author**

1. Is the manuscript technically sound, and do the data support the conclusions?

The manuscript must describe a technically sound piece of scientific research with data that supports the conclusions. Experiments must have been conducted rigorously, with appropriate controls, replication, and sample sizes. The conclusions must be drawn appropriately based on the data presented.

Reviewer #1: Yes

Reviewer #2: Yes

Reviewer #3: Yes

**********

2. Has the statistical analysis been performed appropriately and rigorously?

Reviewer #1: Yes

Reviewer #2: Yes

Reviewer #3: Yes

**********

3. Have the authors made all data underlying the findings in their manuscript fully available?

The PLOS Data policy requires authors to make all data underlying the findings described in their manuscript fully available without restriction, with rare exception (please refer to the Data Availability Statement in the manuscript PDF file). The data should be provided as part of the manuscript or its supporting information, or deposited to a public repository. For example, in addition to summary statistics, the data points behind means, medians and variance measures should be available. If there are restrictions on publicly sharing data—e.g. participant privacy or use of data from a third party—those must be specified.

Reviewer #1: Yes

Reviewer #2: Yes

Reviewer #3: Yes

**********

4. Is the manuscript presented in an intelligible fashion and written in standard English?

PLOS ONE does not copyedit accepted manuscripts, so the language in submitted articles must be clear, correct, and unambiguous. Any typographical or grammatical errors should be corrected at revision, so please note any specific errors here.

Reviewer #1: Yes

Reviewer #2: Yes

Reviewer #3: Yes

**********

5. Review Comments to the Author

Please use the space provided to explain your answers to the questions above. You may also include additional comments for the author, including concerns about dual publication, research ethics, or publication ethics. (Please upload your review as an attachment if it exceeds 20,000 characters)

Reviewer #1: Greetings

Very good work. However, it is recommended to replace the outdated references with more recent studies (published after 2018). In particular, the authors may consider using the following two recent references, which are closely and directly related to the scope and findings of the present study.

Hasan SA, Raoof WM, Ahmed KK. Antibacterial activity of deer musk and Ziziphus spina-christi against carbapebem resis-tant gram negative bacteria isolated from patients with burns and wounds. Regulatory Mechanisms in Biosystems. 2024 Apr 17;15(2):267-78. [DOI: https://doi.org/10.15421/022439]

Hasan SA, Raoof WM, Ahmed KK. FIRST REPORT OF CO-HARBORING BLEOMYCIN RESISTANCE GENE (bleMBL) AND CARBAPENEMASE RESISTANCE GENE (blaNDM-1) KLEBSIELLA PNEUMONIAE IN IRAQ WITH COMPARISON STUDY AMONG THE SENSITIVITY TEST, THE BD PHOENIX CPO DETECT TEST, AND THE RAPIDEC® CARBA NP TEST. Siberian Journal of Life Sciences and Agriculture. 2024 Aug 31;16(4):208-37. [https://doi.org/10.12731/2658-6649-2024-16-4-1249]

Kind regards

Reviewer #2: Dear Editor,

Thank you for inviting me to review this manuscript. I appreciate the opportunity to contribute to the evaluation of this work.

The topic of Multidrug Resistance (MDR) and Carbapenemase Production Among Major Healthcare-Associated Infection (HAI) Pathogens is incredibly important, especially in the context of hospitals, where patients are more vulnerable to infections due to weakened immune systems, invasive procedures, and the high use of antibiotics. It address critical public health issue, it has impact on treatment protocol, improving infection prevention. However, I have noted only a few minor corrections, which are outlined below for the authors.

Title:

• Hyphen between Healthcare and associated, Healthcare-associated

• Omit hyphen from multidrug-resistance

• Please rewrite as “major pathogens causing healthcare-associated infections”

• The title seems too long. Instead “ Multidrug Resistance and Carbapenemase Production Among Major Healthcare-Associated Infection Pathogens in Hospitalized Patients at the University of Gondar Comprehensive Specialized Hospital, Northwest Ethiopia”

Abstract

Method

• Clinical samples (blood, urine, and wound/pus) were aseptically collected and processed following standard microbiological techniques.

• Data were analyzed using SPSS version 27 and a p-value of <0.05 was considered statistically significant.

Result

• What is five to seventeen? You mean years?

You’ve used a margin of error of 4%, but your confidence level is set at 95%, which seems contradictory. You may want to either adjust the margin of error to 5% or increase the confidence level to 96%. (Line 121)

aeruginosa are presented in Fig 1. Where is the figure? I didn’t see it (Line283)

Reviewer #3: Manuscript title: Multidrug-resistance patterns and carbapenemase production in major healthcare-associated infection pathogens among hospitalized patients at the University of Gondar Comprehensive Specialized Hospital, Northwest Ethiopia

Dear editor,

In this manuscript, and based on its contest, the author raised an important issue and tried to address the most global, regional and national sensitive issues, drug resistance. The document (including the introduction, the methods, the discussion…) is well written, well organized, or structured. How ever, the manuscript has a number of issues (comments, and questions) that need to be addressed before published in the journal. Some of the most important comments and questions have been addressed in the reviewer session for the author.

Decision of the reviewer: Minor revision

Comments

Minor

I. As it is an agreed standard, it is better to use scientific names of short forms in the abstracts too, to reduce the number of words and space. E.g. K. pneumonia instead of Klebsiella pneumonia

II. There are a number of textual and grammatical errors, fonts. Better to check thoroughly and consider in your final submission

III. Line 34: structured questionnaire- better to say semi-structured questionnaire

IV. Line 337: Carbapenemase (CP) production was detected in 34 (82.9%) of carbapenem-resistant isolates. Please re-state, as it is not clear.

V. Line 390-391: On your discussion sensitivity testing, and inadequate adherence to antimicrobial stewardship practices [40]. Healthcare-associated infections could be acquired through direct or indirect contact between patients, healthcare workers, visitors, or contaminated objects within hospital environments [54]. As it is not your part better to remove.

Major comments

I. The title seems too specific or too narrow (single study site, specific bacteria…)

II. The result parts of the abstract, lacks clarity as some stated under the question session

III. Your results are good and explanatory, but it seems that you tried to include only tables. Better to consider some figures

IV. Your discussion is fine. However, it is better if you put the implications of the findings.

V. Your conclusion part: “This study revealed a 24.7% prevalence of HAIs and 82.2% rate of MDR among World Health Organization-priority antimicrobial-resistant pathogens, including K. pneumoniae, Acinetobacter spp., and P. aeruginosae.” Is not a good conclusion remark. Better to re-write.

Questions

1. Why did you make a multicenter study? As it was better explanatory and used for generalization.

2. What is especial for your study as there are a number of related previously conducted and published articles in the North eastern and North western Ethiopia including but not limited for:

1. Kemal M, Demeke G, Adugna A, Dilnessa T, Abebaw A, Esmael A. Prevalence, antimicrobial resistance profiles, and determinants of Acinetobacter baumannii and Pseudomonas aeruginosa isolates among nosocomial infection--suspected patients in the northwestern region of Ethiopia. American Journal of Infection Control. 2025 Mar 7.1.

2. Asmare Z, Reta MA, Gashaw Y, Getachew E, Sisay A, Gashaw M, Tamrat E, Kidie AA, Abebe W, Misganaw T, Ashagre A. Antimicrobial resistance profile of Pseudomonas aeruginosa clinical isolates from healthcare-associated infections in Ethiopia: A systematic review and meta-analysis. Plos one. 2024 Aug 13;19(8):e0308946.

3. Mekonnen H, Seid A, Molla Fenta G, Gebrecherkos T. Antimicrobial resistance profiles and associated factors of Acinetobacter and Pseudomonas aeruginosa nosocomial infection among patients admitted at Dessie comprehensive specialized Hospital, North-East Ethiopia. A cross-sectional study. Plos one. 2021 Nov 15;16(11):e0257272.

3. Why you are interested to assess only the three bacterial pathogens?

4. On your abstract, Line 44: what are the number or percentage of Carbapenem resistance pattern of the isolates?

5. For which species of bacteria that Amikacin, chloramphenicol, meropenem, and ciprofloxacin were the most effective antimicrobials?

6. In your sample size calculation, you consider the p = 23.7% from the previous study. However, as I checked, this is only for K. pneumonia. Could you generalize by taking the P from a single etiologic agent, and considering for others?

7. How could you manage for those bacteria that are intrinsically resistance in including and assessing the resistance patterns. E.g. A. baumannii?

8. In your result analysis, did you consider the carbapenem production in the assessment of MDR or exclude them?

**********

6. PLOS authors have the option to publish the peer review history of their article (what does this mean?). If published, this will include your full peer review and any attached files.

If you choose “no”, your identity will remain anonymous but your review may still be made public.

**Do you want your identity to be public for this peer review?** For information about this choice, including consent withdrawal, please see our Privacy Policy.

Reviewer #1: No

Reviewer #2: No

Reviewer #3: No

**********

[NOTE: If reviewer comments were submitted as an attachment file, they will be attached to this email and accessible via the submission site. Please log into your account, locate the manuscript record, and check for the action link "View Attachments". If this link does not appear, there are no attachment files.]

To ensure your figures meet our technical requirements, please review our figure guidelines: https://journals.plos.org/plosone/s/figures

You may also use PLOS’s free figure tool, NAAS, to help you prepare publication quality figures: https://journals.plos.org/plosone/s/figures#loc-tools-for-figure-preparation.

NAAS will assess whether your figures meet our technical requirements by comparing each figure against our figure specifications.

Attachment Submitted filename: Manuscript review for PONE-D-25-63747 Dec 31 2025.docx

# Author comment: Author response to Decision Letter 1

**DOI:** 10.1371/journal.pone.0344280.r002

4 Feb 2026

We sincerely thank and appreciate the Editor and the Reviewers for their constructive and valuable comments and for the time they devoted to reviewing our manuscript. The constructive suggestions have substantially helped us to improve the quality of the manuscript. We have carefully revised the manuscript to address all concerns given by the Editor and Reviewers on manuscript in title of “Multidrug resistance patterns and carbapenemase production among Gram-negative bacteria causing healthcare-associated infections in hospitalized patients at the University of Gondar Comprehensive Specialized Hospital, Northwest Ethiopia” with manuscript ID: PONE-D-25-63747. The authors’ responses to the comments and questions raised by the Editor and Reviewers are highlighted in yellow. Similarly, all important corrections in the manuscript are highlighted in yellow in the revised version ('Revised Manuscript with Track Changes').

Editor comment

1. Please ensure that your manuscript meets PLOS ONE's style requirements, including those for file naming.

2. Please amend the manuscript submission data

3. Please amend your authorship list in your manuscript file to include author Kindu Alem Molla

4. If the reviewer comments include a recommendation to cite specific previously published works, please review and evaluate these publications to determine whether they are relevant and should be cited.

5. The paper requires intensive revision in terms of grammar typographical errors.

6. Essential components such as Conflict of interest, consent for publication, availability of data and material, funding, etc. should be included in the declaration sections of the paper.

7. What has been done for positive cases for the bacteria? It should be mentioned in the ethical consideration section.

8. Properly follow the authors guide for manuscript writing of PloS One.

Authors’ response to Editor’s comment

1. We prepared our manuscript based on author guideline of PLOS ONE. Thus, it meets PLOS ONE's style requirements.

2. We have reviewed and amended the manuscript submission data by checking the title, author names, corresponding author details, abstracts, key words, funding information, ethics approval statement, conflict of interest declaration, and cover letter details.

3. The author Kindu Alem is included in the authorship list of the manuscript

4. The reference suggested by the reviewer has been included and appropriately cited in the manuscript.

5. The manuscript has been carefully reviewed and revised to address the grammatical and topographical errors throughout the text.

6. The manuscript has been revised with the comments given by the Editor to include conflict of interest, consent for publication, availability of data, and funding.

7. For study participants whose samples tested positive for pathogenic bacteria, the results were reported to the attending clinicians, and it is incorporated in the ethical section.

8. The manuscript has been written in strict accordance with the PLOS ONE author guidelines.

Reviewer’s comments

Reviewer #1:

1. Replace the outdated references with more recent studies. In particular, the authors may consider using the following two recent references, which are closely and directly related to the scope and findings of the present study.

Authors’ response to reviewer #1 comments

1. We have included the recent reference commented by the reviewer.

Reviewer #2:

1. Comments on Title:

Hyphen between Healthcare and associated, Healthcare-associated

• Omit hyphen from multidrug-resistance

• Please rewrite as “major pathogens causing healthcare-associated infections”

• The title seems too long.

2. What is five to seventeen? You mean years?

3. You’ve used a margin of error of 4%, but your confidence level is set at 95%, which seems contradictory. You may want to either adjust the margin of error to 5% or increase the confidence level to 96%. (Line 121)

4. P. aeruginosa are presented in Fig 1. Where is the figure? I didn’t see it (Line283)

Authors’ response to reviewer #2 comments and questions

There is already hyphen between healthcare and associated and we have removed hyphen from multidrug-resistance. We have rewritten the title as “Multidrug resistance patterns and carbapenemase production among Gram-negative bacteria causing healthcare-associated infections in hospitalized patients at the University of Gondar Comprehensive Specialized Hospital, Northwest Ethiopia”.

1. The age category was revised from “five to seventeen” to 5-17 years with all related analysis updatee accordingly.

2. 95% confidence level with 4% margin of error is statistically is valid and commonly used in biomedical researches.

3. Although Fig 1. is sited in the manuscript, however, it was submitted as a separate file in accordance with PLOS ONE author guidelines.

Reviewer #3:

I. As it is an agreed standard, it is better to use scientific names of short forms in the abstracts too, to reduce the number of words and space. E.g. K. pneumonia instead of Klebsiella pneumonia

II. There are a number of textual and grammatical errors, fonts. Better to check thoroughly and consider in your final submission.

III. Line 34: structured questionnaire- better to say semi-structured questionnaire

IV. Line 337: Carbapenemase (CP) production was detected in 34 (82.9%) of carbapenem-resistant isolates. Please re-state, as it is not clear.

V. Line 390-391: On your discussion sensitivity testing, and inadequate adherence to antimicrobial stewardship practices [40]. Healthcare-associated infections could be acquired through direct or indirect contact between patients, healthcare workers, visitors, or contaminated objects within hospital environments [54]. As it is not your part better to remove.

VI. The title seems too specific or too narrow (single study site, specific bacteria

VII. The result parts of the abstract, lacks clarity as some stated under the question session

VIII. Your results are good and explanatory, but it seems that you tried to include only tables. Better to consider some figures

IX. Your discussion is fine. However, it is better if you put the implications of the findings.

X. Your conclusion part: “This study revealed a 24.7% prevalence of HAIs and 82.2% rate of MDR among World Health Organization-priority antimicrobial-resistant pathogens, including K. pneumoniae, Acinetobacter spp., and P. aeruginosa.” Is not a good conclusion remark. Better to re-write.

1. Why did you make a multicenter study? As it was better explanatory and used for generalization.

2. What is especial for your study as there are a number of related previously conducted and published articles in the North eastern and North western Ethiopia

3. Why you are interested to assess only the three bacterial pathogens?

4. On your abstract, Line 44: what are the number or percentage of Carbapenem resistance pattern of the isolates?

5. For which species of bacteria that Amikacin, chloramphenicol, meropenem, and ciprofloxacin were the most effective antimicrobials?

6. In your sample size calculation, you consider the p = 23.7% from the previous study. However, as I checked, this is only for K. pneumonia. Could you generalize by taking the P from a single etiologic agent, and considering for others?

7. How could you manage for those bacteria that are intrinsically resistance in including and assessing the resistance patterns. E.g. A. baumannii?

8. In your result analysis, did you consider the carbapenem production in the assessment of MDR or exclude them?

Authors’ response to reviewer #3 comments and questions

I. In the abstract, the first mention of each organism uses the full scientific name (Klebsiella pneumoniae and Pseudomonas aeruginosa) which is recommended by most journal including PLOS ONE for clarity of readers, followed by the appropriate abbreviations (K. pneumoniae and P. aeruginosa) in subsequent mentions.

II. Textual and grammatical error, as well as font size inconsistency, have been corrected in the final manuscript. However, a small font size was used for large tables and figures, which is permitted by the PLOS ONE journal guidelines.

III. Line 34: structured questionnaire is rewritten in semi-structured questionnaire.

IV. We have included a sentence to improve clarity by specifying the total number of carbapenem-resistant isolates and clearly distinguishing carbapenemase producing from non-producing isolates.

V. In our discussion part we have not removed healthcare workers, or contaminated objects within hospital environments since the contact between health workers, patients and hospital environment plays a significant role in the transmission of healthcare associated infections in hospitalized patients.

VI. The title of the manuscript is specific which helps to clearly reflect the study setting, patient population, and pathogens investigated.

VII. We have addressed the reviewer’s comments regarding the lack of clarity on the results section of the abstract.

VIII. We have added three figures presenting antimicrobial resistance patterns of (a) K. pneumoniae, (b) Acinetobacter spp., and (c) P. aeruginosa. Although they are combined under a single figure title, each panel clearly represents the respective pathogen.

IX. We have addressed the implication of our findings in the discussion, emphasizing their relevance for clinical practice and infection control.

X. We have revised the conclusion part to avoid reporting specific figures and to provide a more interpretive and meaningful summary.

1. Our study was not designed as a multicenter study, rather, it was a hospital based cross-sectional study at one specialized hospital. It was clearly indicated in the methods part of the manuscript

2. There are very few studies conducted in this study are concerning K. pneumoniae, Acinetobacter spp., and P. aeruginosa in relation to healthcare-associated infections caused by these three bacteria, especially in the study area, Gondar. Even, the very few studies reported does not include K. pneumoniae, which is one of the major pathogens that cause healthcare-associated infections. This gap initiates us to focus on these three bacteria which are the major healthcare-associated infections especially in intensive care units.

3. The reason why we focused on the three bacteria, Klebsiella pneumoniae, Acinetobacter baumannii, and Pseudomonas aeruginosa, is because they are the most prevalent causes of healthcare-associated infections, and they are recognized as WHO priority pathogens due to their high level of MDR and carbapenem resistance. These pathogens also highly associated with prolonged hospitalization and limited treatment options.

4. On line 44 on abstract part, we have reported the percentage of carbapenemase production in K. pneumoniae, Acinetobacter spp., and P. aeruginosa. However, the percentage of carbapenem resistance was reported on the result part Figure 1 which was submitted separately during submission with carbapenem resistance pattern of K. pneumoniae, Acinetobacter spp., and P. aeruginosa were 35.2%, 39.1%, and 29.2%, respectively. However, we have reported on result section page 13 line 295 and 296 of the revised manuscript.

5. Amikacin, chloramphenicol, and meropenem were effective for K. pneumoniae. For Acinetobacter spp., amikacin and meropenem were effective antibiotics. In P. aeruginosa, amikacin, ciprofloxacin, and meropenem were effective. Chloramphenicol was not tested against Acinetobacter spp. and P. aeruginosa because it is not recommended by CLSI guidelines for these pathogens.

6. The prevalence value of 23.7% was selected from a previous study on Klebsiella pneumoniae because it represents the highest reported prevalence among the target pathogens (K. pneumoniae, Acinetobacter spp., and Pseudomonas aeruginosa) in comparable settings. Applying the maximum prevalence provides or it yields a large sample size and ensures adequate power to detect resistance pattern across all included pathogens.

7. For organisms in which intrinsically resistance (e.g., A. baumannii), antibiotics to which the species is intrinsically resistant were excluded from resistance rate determination and MDR classification, in accordance CLSI guideline.

8. Carbapenemase production was analyzed separately as a resistance mechanism, not as MDR. MDR classification was based on antimicrobial susceptibility testing results across different antibiotic classes.

Attachment Submitted filename: Response to Reviewers.docx

# Peer review: Decision Letter 1

**DOI:** 10.1371/journal.pone.0344280.r003

**Authors:** Tebelay Dilnessa

10 Feb 2026

PONE-D-25-63747R1

Multidrug resistance patterns and carbapenemase production among Gram-negative bacteria causing healthcare-associated infections in hospitalized patients at the University of Gondar Comprehensive Specialized Hospital, Northwest Ethiopia

PLOS One

Dear Dr. Molla,

Thank you for submitting your manuscript to PLOS ONE. After careful consideration, we feel that it has merit but does not fully meet PLOS ONE’s publication criteria as it currently stands. Therefore, we invite you to submit a revised version of the manuscript that addresses the points raised during the review process.

Please submit your revised manuscript by Mar 27 2026 11:59PM. If you will need more time than this to complete your revisions, please reply to this message or contact the journal office at plosone@plos.org. When you're ready to submit your revision, log on to https://www.editorialmanager.com/pone/ and select the 'Submissions Needing Revision' folder to locate your manuscript file.

Please include the following items when submitting your revised manuscript:

- A letter that responds to each point raised by the academic editor and reviewer(s). You should upload this letter as a separate file labeled 'Response to Reviewers'.
- A marked-up copy of your manuscript that highlights changes made to the original version. You should upload this as a separate file labeled 'Revised Manuscript with Track Changes'.
- An unmarked version of your revised paper without tracked changes. You should upload this as a separate file labeled 'Manuscript'.

If you would like to make changes to your financial disclosure, please include your updated statement in your cover letter. Guidelines for resubmitting your figure files are available below the reviewer comments at the end of this letter.

If applicable, we recommend that you deposit your laboratory protocols in protocols.io to enhance the reproducibility of your results. Protocols.io assigns your protocol its own identifier (DOI) so that it can be cited independently in the future. For instructions see: https://journals.plos.org/plosone/s/submission-guidelines#loc-laboratory-protocols. Additionally, PLOS ONE offers an option for publishing peer-reviewed Lab Protocol articles, which describe protocols hosted on protocols.io. Read more information on sharing protocols at https://plos.org/protocols?utm_medium=editorial-email&utm_source=authorletters&utm_campaign=protocols.

We look forward to receiving your revised manuscript.

Kind regards,

Tebelay Dilnessa, MSc

Academic Editor

PLOS One

**Journal Requirements:**

1. If the reviewer comments include a recommendation to cite specific previously published works, please review and evaluate these publications to determine whether they are relevant and should be cited. There is no requirement to cite these works unless the editor has indicated otherwise.

2. Please review your reference list to ensure that it is complete and correct. If you have cited papers that have been retracted, please include the rationale for doing so in the manuscript text, or remove these references and replace them with relevant current references. Any changes to the reference list should be mentioned in the rebuttal letter that accompanies your revised manuscript. If you need to cite a retracted article, indicate the article’s retracted status in the References list and also include a citation and full reference for the retraction notice.

**Additional Editor Comments:**

**Abstract**

- Line 24: **Background**, not introduction
- Line 28-31: The objective part lacks the main objective, ‘carbapenemase production.’ Even the general objective should be parallel with the title of the manuscript. Therefore, it requires revision.
- To which media were the specimens inoculated?
- The analysis part also should be incorporated with Bivariate/multivariate logistic regression
- Line 41: Significant associated factors included………….
- Line 48: This study showed……
- Line 52: carbapenemase production
- Lines 94 and 95: Therefore, the aim of this study was to determine the prevalence and AMR patterns and associated factors…………..

**Materials and methods**

- Line 108 and 109: Now, the hospital serves more than 5 million people in the catchment area [18].
- Please make UoGCSH rather than UGCSH throughout the document
- Line 119: **Sample size determination and sampling technique**
- Line 120: The sample size was………………..
- Line 219: **Data quality assurance**
- **Results**
- Line 256: **Isolation rate of *K. pneumoniae, Acinetobacter* spp., and *P. aeruginosa***
- Line 289 and 290: Remove the following sentence ‘The antimicrobial susceptibility patterns of the isolated *K. pneumoniae, Acinetobacter* spp., and *P. aeruginosa* are presented in Fig 1.’ and add (Fig.1) at the end of this paragraph in bracket. Again, the caption for this figure should be placed below this citation, i.e. Fig 1. Antimicrobial resistance patterns of (a*) K. pneumoniae,* (b) *Acinetobacter spp.,* and (c) *P. aeruginosa* among patients suspected of having HAIs at UGCSH, Gondar, Northwest Ethiopia.
- Line 313**:** Remove ‘The overall MDR prevalence among all isolates was 82.2% (97/118; 95% CI: 75.3-89.1)’ and also from Figure 7 of the last row. It is impossible to calculate/determine overall MDR for more than one type of bacteria.
- **References**
- The name of the journal and bacteria name should be italized.
- Line 608: CLSI. Performance Standards for Antimicrobial…………………

[Note: HTML markup is below. Please do not edit.]

Reviewers' comments:

Reviewer's Responses to Questions

**Comments to the Author**

1. If the authors have adequately addressed your comments raised in a previous round of review and you feel that this manuscript is now acceptable for publication, you may indicate that here to bypass the “Comments to the Author” section, enter your conflict of interest statement in the “Confidential to Editor” section, and submit your "Accept" recommendation.

Reviewer #2: All comments have been addressed

Reviewer #3: All comments have been addressed

**********

2. Is the manuscript technically sound, and do the data support the conclusions?

The manuscript must describe a technically sound piece of scientific research with data that supports the conclusions. Experiments must have been conducted rigorously, with appropriate controls, replication, and sample sizes. The conclusions must be drawn appropriately based on the data presented.

Reviewer #2: Yes

Reviewer #3: Yes

**********

3. Has the statistical analysis been performed appropriately and rigorously?

Reviewer #2: Yes

Reviewer #3: Yes

**********

4. Have the authors made all data underlying the findings in their manuscript fully available?

The PLOS Data policy requires authors to make all data underlying the findings described in their manuscript fully available without restriction, with rare exception (please refer to the Data Availability Statement in the manuscript PDF file). The data should be provided as part of the manuscript or its supporting information, or deposited to a public repository. For example, in addition to summary statistics, the data points behind means, medians and variance measures should be available. If there are restrictions on publicly sharing data—e.g. participant privacy or use of data from a third party—those must be specified.

Reviewer #2: Yes

Reviewer #3: Yes

**********

5. Is the manuscript presented in an intelligible fashion and written in standard English?

PLOS ONE does not copyedit accepted manuscripts, so the language in submitted articles must be clear, correct, and unambiguous. Any typographical or grammatical errors should be corrected at revision, so please note any specific errors here.

Reviewer #2: Yes

Reviewer #3: Yes

**********

6. Review Comments to the Author

Please use the space provided to explain your answers to the questions above. You may also include additional comments for the author, including concerns about dual publication, research ethics, or publication ethics. (Please upload your review as an attachment if it exceeds 20,000 characters)

Reviewer #2: (No Response)

Reviewer #3: All the comments have been addressed. Moreover, all the questions have been clearly justified. The authors raised an important topic on Multidrug resistance patterns and carbapenemase production among Gram-negative

bacteria causing healthcare-associated infections in hospitalized patients. The objectives are clearly stated. No more comments. All questions and comments are addressed

**********

7. PLOS authors have the option to publish the peer review history of their article (what does this mean?). If published, this will include your full peer review and any attached files.

If you choose “no”, your identity will remain anonymous but your review may still be made public.

**Do you want your identity to be public for this peer review?** For information about this choice, including consent withdrawal, please see our Privacy Policy.

Reviewer #2: No

Reviewer #3: **Yes:**Dessie Tegegne

**********

[NOTE: If reviewer comments were submitted as an attachment file, they will be attached to this email and accessible via the submission site. Please log into your account, locate the manuscript record, and check for the action link "View Attachments". If this link does not appear, there are no attachment files.]

To ensure your figures meet our technical requirements, please review our figure guidelines: https://journals.plos.org/plosone/s/figures

You may also use PLOS’s free figure tool, NAAS, to help you prepare publication quality figures: https://journals.plos.org/plosone/s/figures#loc-tools-for-figure-preparation.

NAAS will assess whether your figures meet our technical requirements by comparing each figure against our figure specifications.

# Author comment: Author response to Decision Letter 2

**DOI:** 10.1371/journal.pone.0344280.r004

17 Feb 2026

We sincerely thank and appreciate the Editor and the Reviewers for their constructive and valuable comments and for the time they devoted to reviewing our manuscript. The constructive suggestions have substantially helped us to improve the quality of the manuscript. We have carefully revised the manuscript to address all concerns given by the Editor and Reviewers on manuscript in title of “Multidrug resistance patterns and carbapenemase production among Gram-negative bacteria causing healthcare-associated infections in hospitalized patients at the University of Gondar Comprehensive Specialized Hospital, Northwest Ethiopia” with manuscript ID: PONE-D-25-63747. The authors’ responses to the comments and questions raised by the Editor and Reviewers are responded accordingly. Similarly, all important corrections in the manuscript are highlighted in yellow in the revised version ('Revised Manuscript with Track Changes').

Journal Requirements

1. If the reviewer comments include a recommendation to cite specific previously published works, please review and evaluate these publications to determine whether they are relevant and should be cited. There is no requirement to cite these works unless the editor has indicated otherwise.

Response: Thank you for the comment. Reviewer 1 recommended that we consider two references, including (1) Hasan SA, Raoof WM, Ahmed KK. Antibacterial activity of deer musk and Ziziphus spina-christi against carbapebem resis-tant gram negative bacteria isolated from patients with burns and wounds. Regulatory Mechanisms in Biosystems. 2024 Apr 17;15(2):267-78. [DOI: https://doi.org/10.15421/022439] and (2) Hasan SA, Raoof WM, Ahmed KK. FIRST REPORT OF CO-HARBORING BLEOMYCIN RESISTANCE GENE (bleMBL) AND CARBAPENEMASE RESISTANCE GENE (blaNDM-1) KLEBSIELLA PNEUMONIAE IN IRAQ WITH COMPARISON STUDY AMONG THE SENSITIVITY TEST, THE BD PHOENIX CPO DETECT TEST, AND THE RAPIDEC® CARBA NP TEST. Siberian Journal of Life Sciences and Agriculture. 2024 Aug 31;16(4):208-37. [https://doi.org/10.12731/2658-6649-2024-16-4-1249]. After careful evaluation, we found that the first reference is relevant to our study and has been incorporated into the revised manuscript. However, the second reference is not directly aligned with the scope and objectives of our work.

2. Please review your reference list to ensure that it is complete and correct.

Response: Thank you for the comment. We have carefully reviewed and updated the reference lists to insure its completeness and correctness. References that are published before 2018 have been removed where appropriate, however, seminal or foundational studies essential to the context of our have been retained. works are included. Additionally, we confirm that no retracted articles are cited in the manuscript.

Additional Editor Comments

Abstract

o Line 24: Background, not introduction

o Line 28-31: The objective part lacks the main objective, ‘carbapenemase production.’ Even the general objective should be parallel with the title of the manuscript. Therefore, it requires revision.

o To which media were the specimens inoculated?

o The analysis part also should be incorporated with Bivariate/multivariate logistic regression

o Line 41: Significant associated factors included………….

o Line 48: This study showed……

o Line 52: carbapenemase production

o

o Lines 94 and 95: Therefore, the aim of this study was to determine the prevalence and AMR patterns and associated factors…………..

Materials and methods

� Line 108 and 109: Now, the hospital serves more than 5 million people in the catchment area [18].

� Please make UoGCSH rather than UGCSH throughout the document

� Line 119: Sample size determination and sampling technique

� Line 120: The sample size was………………..

� Line 219: Data quality assurance

� Results

� Line 256: Isolation rate of K. pneumoniae, Acinetobacter spp., and P. aeruginosa

� Line 289 and 290: Remove the following sentence ‘The antimicrobial susceptibility patterns of the isolated K. pneumoniae, Acinetobacter spp., and P. aeruginosa are presented in Fig 1.’ and add (Fig.1) at the end of this paragraph in bracket. Again, the caption for this figure should be placed below this citation, i.e. Fig 1. Antimicrobial resistance patterns of (a) K. pneumoniae, (b) Acinetobacter spp., and (c) P. aeruginosa among patients suspected of having HAIs at UGCSH, Gondar, Northwest Ethiopia.

� Line 313: Remove ‘The overall MDR prevalence among all isolates was 82.2% (97/118; 95% CI: 75.3-89.1)’ and also from Figure 7 of the last row. It is impossible to calculate/determine overall MDR for more than one type of bacteria.

� References

� The name of the journal and bacteria name should be italized.

� Line 608: CLSI. Performance Standards for Antimicrobial…………………

Responses to Editor Comments for abstract part

o Thank you for the comment. In the abstract part, the heading “Introduction” is replaced with “Background” as suggested.

o Thank you for the constructive comment. The objective is corrected by incorporating carbapenemase production as follows “The aim of this study was to determine the prevalence, associated risk factors, antimicrobial resistance patterns, and carbapenemase production of K. pneumoniae, Acinetobacter spp., and P. aeruginosa among hospitalized patients suspected of healthcare-associated infections at the University of Gondar Comprehensive Specialized Hospital, Northwest Ethiopia.”

o Thank you for the valuable comment. The specimens were inoculated onto MacConkey, blood, and cysteine lactose electrolyte-deficient agar, as clearly stated in the revised manuscript.

o Thank you for the helpful comment. Bivariate and multivariate logistic regression analyzes have been incorporated in the method section of the abstract.

o Thank you for the valuable comment. The phrase “Significant risk factors” has been revised to “Significant associated factors” as recommended.

o Thank you for the constructive comment. The phrase “our study shows” has been revised into “This study showed” as suggested.

o Thank you for the comment. The phrase “carbapenemase production” is incorporated into the conclusion section of the abstract in the revised manuscript.

o Thank you for the comment. In lines 94 and 95 of the introduction, the word “Thus” is replaced with “Therefore” as suggested.

Responses to Editor Comments for Materials and Methods

o Thank you for the valuable comment. In the revised manuscript, line 104 has been corrected to read: “Now, the hospital serves more than 5 million people in the catchment area”, with a comma added after “Now” as recommended.

o Thank you for the comment. All occurrence of “UGCSH” have been corrected to “UoGCSH” throughout the revised manuscript.

o Thank you for the valuable comment. On line 114 of the revised manuscript “Sample size and sampling procedures” has been corrected to “Sample size determination and sampling techniques” as suggested.

o Thank you for the comment. On line 115 of the revised manuscript, “Sample size was” has been corrected to “The sample size was” as recommended.

o Thank you for the comment. On line 210 of the revised manuscript, “Quality assurance” has been corrected to “Data quality assurance” as suggested.

Responses to Editor Comments for Result part

o Thank you for the comment. On line 253 of the revised manuscript, the heading “Isolation rate of K. pneumoniae, Acinetobacter spp., and P. aeruginosa in healthcare-associated infections” has been corrected to “Isolation rate of K. pneumoniae, Acinetobacter spp., and P. aeruginosa” as recommended.

o Thank you for the constructive comment. On lines 289 and 290 of previous unrevised manuscript, the sentence “The antimicrobial susceptibility patterns of the isolated K. pneumoniae, Acinetobacter spp., and P. aeruginosa are presented in Fig 1.” Has been removed and “(Fig 1) has been added at the end of the paragraph. Additionally, the figure caption has been placed below the citation as recommended. “

Fig 1. Antimicrobial resistance patterns of (a) K. pneumoniae, (b) Acinetobacter spp., and (c) P. aeruginosa among patients suspected of having HAIs at UGCSH, Gondar, Northwest Ethiopia. HAIs, healthcare-associated infections; UoGCSH, University of Gondar Comprehensive Specialized Hospital; AMC, amoxicillin-clavulanic acid; PIP, piperacillin; TZP, piperacillin-tazobactam; CRO, ceftriaxone; CAZ, ceftazidime; CTX, cefotaxime; MER, meropenem; IMP, imipenem; AMK, amikacin; GEN, gentamicin; TOB, tobramycin; TET, tetracycline; CIP, ciprofloxacin; COT, co-trimoxazole; CHL, chloramphenicol

o Thank you for the constructive comment. On line 313 of the unrevised manuscript, the sentence “The overall MDR prevalence among all isolates was 82.2% (97/118; 95% CI: 75.3-89.1) has been removed, and the corresponding value has also been removed from the last row of Figure 7, since calculating an overall MDR across multiple bacterial species is not appropriate.

Responses to Editor Comments to References Part

o Thank you for the comment. In all references, the journal names and bacterial scientific names have been italicized as required.

o Thank you for the comment. On line 608 of the previous unrevised manuscript, the reference has been corrected from “CLSI, Performance Standards for Antimicrobial Susceptibility Testing, 34th Edition. 2024. 1-382” to “CLSI. Performance Standards for Antimicrobial Susceptibility Testing, 34th Edition. 2024. 1-382” by removing the comma and adding a full stop after “CLSI” as suggested.

Attachment Submitted filename: Response_to_Reviewers_auresp_2.docx

# Editor report: Decision Letter 2

**DOI:** 10.1371/journal.pone.0344280.r005

**Authors:** Tebelay Dilnessa

19 Feb 2026

Multidrug resistance patterns and carbapenemase production among Gram-negative bacteria causing healthcare-associated infections in hospitalized patients at the University of Gondar Comprehensive Specialized Hospital, Northwest Ethiopia

PONE-D-25-63747R2

Dear Dr. Molla,

We’re pleased to inform you that your manuscript has been judged scientifically suitable for publication and will be formally accepted for publication once it meets all outstanding technical requirements.

Within one week, you’ll receive an e-mail detailing the required amendments. When these have been addressed, you’ll receive a formal acceptance letter and your manuscript will be scheduled for publication.

An invoice will be generated when your article is formally accepted. Please note, if your institution has a publishing partnership with PLOS and your article meets the relevant criteria, all or part of your publication costs will be covered. Please make sure your user information is up-to-date by logging into Editorial Manager at Editorial Manager® and clicking the ‘Update My Information' link at the top of the page. For questions related to billing, please contact billing support.

If your institution or institutions have a press office, please notify them about your upcoming paper to help maximize its impact. If they’ll be preparing press materials, please inform our press team as soon as possible -- no later than 48 hours after receiving the formal acceptance. Your manuscript will remain under strict press embargo until 2 pm Eastern Time on the date of publication. For more information, please contact onepress@plos.org.

Kind regards,

Tebelay Dilnessa, MSc

Academic Editor

PLOS One

Additional Editor Comments (optional):

Reviewers' comments:

# Editor report: Acceptance letter

**DOI:** 10.1371/journal.pone.0344280.r006

**Authors:** Tebelay Dilnessa

PONE-D-25-63747R2

PLOS One

Dear Dr. Molla,

I'm pleased to inform you that your manuscript has been deemed suitable for publication in PLOS One. Congratulations! Your manuscript is now being handed over to our production team.

At this stage, our production department will prepare your paper for publication. This includes ensuring the following:

* All references, tables, and figures are properly cited

* All relevant supporting information is included in the manuscript submission,

* There are no issues that prevent the paper from being properly typeset

You will receive further instructions from the production team, including instructions on how to review your proof when it is ready. Please keep in mind that we are working through a large volume of accepted articles, so please give us a few days to review your paper and let you know the next and final steps.

Lastly, if your institution or institutions have a press office, please let them know about your upcoming paper now to help maximize its impact. If they'll be preparing press materials, please inform our press team within the next 48 hours. Your manuscript will remain under strict press embargo until 2 pm Eastern Time on the date of publication. For more information, please contact onepress@plos.org.

You will receive an invoice from PLOS for your publication fee after your manuscript has reached the completed accept phase. If you receive an email requesting payment before acceptance or for any other service, this may be a phishing scheme. Learn how to identify phishing emails and protect your accounts at https://explore.plos.org/phishing.

If we can help with anything else, please email us at customercare@plos.org.

Thank you for submitting your work to PLOS ONE and supporting open access.

Kind regards,

PLOS ONE Editorial Office Staff

on behalf of

Dr. Tebelay Dilnessa

Academic Editor

PLOS One
